# Changes of 5-hydroxymethylcytosine distribution during myeloid and lymphoid differentiation of CD34+ cells

**DOI:** 10.1186/s13072-016-0070-8

**Published:** 2016-05-31

**Authors:** Xavier Tekpli, Alfonso Urbanucci, Adnan Hashim, Cathrine B. Vågbø, Robert Lyle, Marianne K. Kringen, Anne Cathrine Staff, Ingunn Dybedal, Ian G. Mills, Arne Klungland, Judith Staerk

**Affiliations:** Nordic European Molecular Laboratory (EMBL) Partnership, Centre for Molecular Medicine Norway, University of Oslo, Blindern, P.O. Box 1137, 0318 Oslo, Norway; Department of Molecular Oncology, Institute for Cancer Research, Oslo University Hospital, Oslo, Norway; Department of Cancer Research and Molecular Medicine, Norwegian University of Science and Technology, Trondheim, Norway; Department of Medical Genetics, University of Oslo, Oslo University Hospital, Oslo, Norway; Department of Pharmacology, Oslo University Hospital, Ullevål, Oslo, Norway; Department of Obstetrics and Gynecology, Oslo University Hospital, University of Oslo, Oslo, Norway; Department of Haematology, Oslo University Hospital, Oslo, Norway; PCUK Movember Centre of Excellence, CCRCB, Queen’s University, Belfast, UK; Clinic for Diagnostics and Intervention, Institute of Medical Microbiology, Oslo University Hospital, University of Oslo, Oslo, Norway; Norwegian Center for Stem Cell Research, University of Oslo, Oslo, Norway; Department of Genetics, Institute for Cancer Research, Oslo University Hospital - The Norwegian Radium Hospital, Oslo, Norway

**Keywords:** 5-Hydroxymethylcytosine, Epigenetics, Hematopoiesis, RUNX, FLI1

## Abstract

**Background:**

Hematopoietic stem cell renewal and differentiation are regulated through epigenetic processes. The conversion of 5-methylcytosine into 5-hydroxymethylcytosine (5hmC) by ten-eleven-translocation enzymes provides new insights into the epigenetic regulation of gene expression during development. Here, we studied the potential gene regulatory role of 5hmC during human hematopoiesis.

**Results:**

We used reduced representation of 5-hydroxymethylcytosine profiling (RRHP) to characterize 5hmC distribution in CD34+ cells, CD4+ T cells, CD19+ B cells, CD14+ monocytes and granulocytes. In all analyzed blood cell types, the presence of 5hmC at gene bodies correlates positively with gene expression, and highest 5hmC levels are found around transcription start sites of highly expressed genes. In CD34+ cells, 5hmC primes for the expression of genes regulating myeloid and lymphoid lineage commitment. Throughout blood cell differentiation, intragenic 5hmC is maintained at genes that are highly expressed and required for acquisition of the mature blood cell phenotype. Moreover, in CD34+ cells, the presence of 5hmC at enhancers associates with increased binding of RUNX1 and FLI1, transcription factors essential for hematopoiesis.

**Conclusions:**

Our study provides a comprehensive genome-wide overview of 5hmC distribution in human hematopoietic cells and new insights into the epigenetic regulation of gene expression during human hematopoiesis.

**Electronic supplementary material:**

The online version of this article (doi:10.1186/s13072-016-0070-8) contains supplementary material, which is available to authorized users.

## Background

Epigenetic regulation of gene expression plays an important role during stem cell renewal and cell fate determination [1]. Ten-eleven-translocation (TET) DNA dioxygenases catalyze the hydroxylation of 5-methylcytosine (5mC) into 5hmC [2]. Apart from being an intermediate during the DNA demethylation process, 5hmC may function as a stable epigenetic mark with regulatory function [3], a notion supported by studies showing that TET enzymes and 5hmC are important for early embryonic development [2, 4].

Hematopoiesis, the lifelong formation of blood cells, is ensured by the renewal of hematopoietic stem cells and their differentiation into mature cell types. Mice lacking TET2 are characterized by pleiotropic alterations of progenitor and mature hematopoietic cells, indicating that TET2 is an important regulator of murine blood cell development [5, 6]. Moreover, inactivating mutations of TET2 are found in various human blood disorders [7]. Recent studies mapping hydroxymethylated regions in blood cells indicated a role of 5hmC during erythropoiesis [8] as well as T cell and B cell differentiation [9, 10]. However, these reports used enrichment-based methods to detect 5hmC and focused on a specific lineage.

To systematically study the role of 5hmC during human hematopoiesis and assess the possible gene regulatory role of 5hmC during lymphoid and myeloid differentiation, we used a single-base resolution method, reduced representation of 5-hydroxymethylcytosine profiling (RRHP) [11]. We mapped 5hmC in CD34+ and mature peripheral blood cells (CD4+ T cells, CD19+ B cells, CD14+ monocytes and granulocytes). By integrating 5hmC profiles with gene expression analysis, histone modifications as well as transcription factor (TF) binding profiles we provide molecular evidence that 5hmC promotes the expression of key genes important for the differentiation of CD34+ cells into either lymphoid or myeloid lineage.

## Results

### Genome-wide distribution and quantification of 5hmC in hematopoietic cells

**Fig. 1:**
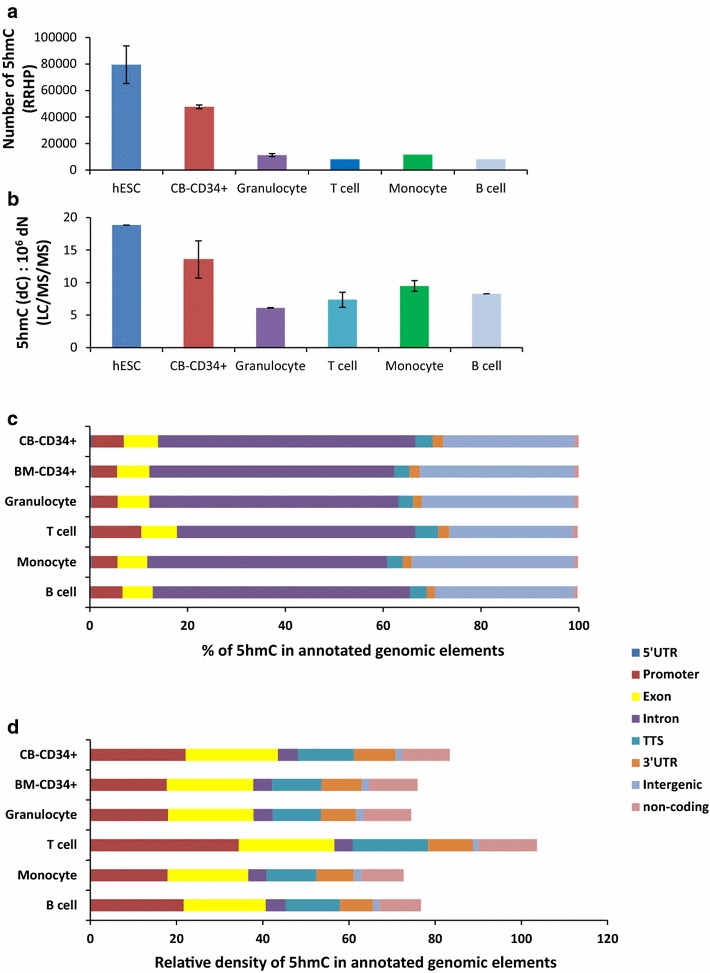
5hmC levels and distribution in hematopoietic cells. **a** Proportion of hydroxymethylated sites in the indicated cell types. The number of 5hmC sites for each cell type was obtained following RRHP data analysis; *n* = 2 for hESC, CB-CD34+ cells and granulocytes; *n* = 1 for CD4+ T cells, CD19+ B cells and CD14+ monocytes. The mean numbers ± SEM are presented. **b** Quantification of 5hmC *per* 1 × 10^6^ nucleotide DNA in hESC (*n* = 1), CB-CD34+ (*n* = 2), CD4+ T cells (*n* = 3), CD19+ B cells (*n* = 1), CD14+ monocytes (*n* = 3) and granulocytes (*n* = 3). Cells were harvested, DNA isolated, hydrolyzed and analyzed for 5hmC using liquid chromatography tandem mass spectrometry (LC/MS/MS). 5hmC levels are normalized relative to 10^6^ nucleosides. Data shown are the mean ± SEM. **c** Percentage of 5hmC in annotated genomic elements for each indicated cell type. Promoters were defined −1-kb upstream to +100-bp downstream of the transcription start site (TSS); transcription termination site (TTS) indicates a region −100-bp upstream to +1-kb downstream of the TTS. *UTR* untranslated region. **d** Relative density of 5hmC in annotated genomic elements for each indicated cell type. The relative density of 5hmC reflects the number of 5hmC encompassed in annotated genomic elements normalized to 10^5^ base pair of the respective element and to a fixed number of 10^5^ 5hmC sites

We used RRHP analysis to quantify and assess the distribution of 5hmC-positive CCGG sites in hESC (*n* = 2), umbilical cord blood (*n* = 2) and bone marrow (*n* = 1) CD34+ cells (CB-CD34+ and BM-CD34+), as well as CD4+ T cells, CD19+ B cells, CD14+ monocytes (*n* = 1 for each) and granulocytes (*n* = 2) (Fig. 1a; Additional file 1: Table S1). We found a strong correlation in replicated samples (hESC, CB-CD34+ and granulocytes) with Pearson’s correlation *r* > 0.75 and *p* < 0.05 (Additional file 1: Table S1). We first compared 5hmC quantification in the different blood cell types using RRHP (Fig. 1a) and liquid chromatography tandem mass spectrometry (LC/MS/MS) (Fig. 1b). We included human embryonic stem cells (hESC) as reference cell type in our analysis since 5hmC has been well characterized in those cells and it is known that pluripotent cells contain high levels of 5hmC [12, 13].

Both methods, RRHP and LC/MS/MS, showed that higher 5hmC levels were found in pluripotent (hESC) cells and multipotent (CD34+) cells compared to mature blood cell types (Fig. 1a, b). This was in contrast to 5mC levels measured by LC/MS/MS, which did not change between the different cell types (Additional file 2: Fig. S1). We next characterized the distribution of 5hmC in annotated elements in the different blood cell types and found that 78–82 % of 5hmC sites localized in introns and intergenic regions (Fig. 1c). Since introns and intergenic regions represent 80 % of the human genome, it was expected that most of the 5hmC sites will fall within these genomic elements. We therefore determined the relative density of 5hmC sites in annotated genomic elements (Fig. 1d) and found high 5hmC density in exons and promoter, indicating that the presence of 5hmC is particularly abundant around annotated genes. Notably, T lymphocytes showed higher 5hmC density in promoters than other cell types, possibly reflecting an important role for 5hmC at promoters in T cells. Gene ontology (GO) analysis of genes with hydroxymethylated promoter in T cells showed a marked enrichment for biological processes related to cell death and T cell regulation (*p* < 10^−6^), as well as pathways related to T cell receptor signaling (*p* < 10^−4^).

Taken together, we found high 5hmC density in gene bodies and showed that 5hmC levels decrease during cell differentiation.

### 5hmC enrichment at gene bodies positively correlates with gene expression

To assess the potential gene regulatory role of 5hmC during human hematopoiesis, we integrated 5hmC profiles with measurement of gene expression by RNA-seq and genome-wide mapping of histone modifications using publically available ChIP-seq data sets.

**Fig. 2:**
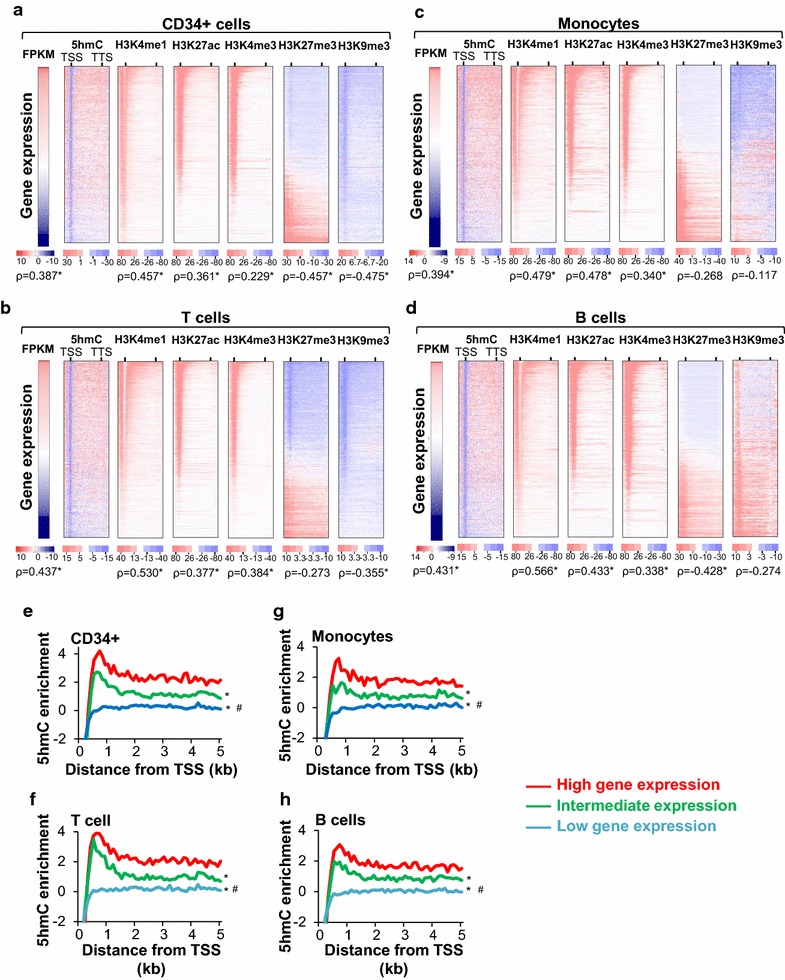
5hmC enrichment in gene bodies positively correlates with gene expression and active chromatin. Heatmaps showing the enrichment of the indicated epigenetic mark −1.5-kb upstream of the TSS to +1.5-kb downstream of the TTS in all annotated human genes ordered by decreasing expression (log2-transformed FPKM values) in CD34+ (**a**), T cells (**b**), monocytes (**c**) and B cells (**d**). The Spearman rank (*ρ*) correlation coefficient below the heatmaps indicates the correlation between gene body 5hmC levels and gene expression (log2 FPKM) or a specific histone modification (exact permutation test for testing the null hypothesis of no correlation, two-tailed, *p < 5 × 10^−20^). Averaged 5hmC enrichment in CD34+ (**e**), T cells (**f**), monocytes (**g**) and B cells (**h**) 5-kb downstream of the TSS of genes expressed at high, low or intermediate level. Genes were divided into tertiles based on expression levels and averaged 5hmC enrichment 5-kb downstream of the TSS. Differences in 5hmC enrichment between the tertiles were assessed using the Kolmogorov–Smirnov test (*; ^#^significant differences in 5hmC enrichment with *D* > 0.45 and *p* < 10^−10^. *High versus intermediate or low gene expression; ^#^intermediate versus low gene expression)

We characterized 5hmC distribution around annotated genes. Figure 2 shows the enrichment of 5hmC −1.5-kb upstream of the transcription start site (TSS) to +1.5-kb downstream of the transcription termination site (TTS). We found that gene body 5hmC correlated positively with gene expression in all analyzed blood cell types (Fig. 2a–d). Specifically, when we categorized 5hmC enrichment 5-kb downstream the TSS, we confirmed that low expressed genes contained low 5hmC levels, while highly expressed genes showed significantly higher 5hmC levels (Fig. 2e–h). Interestingly, 5hmC decreases especially at TSS of highly expressed genes [indicated by the blue line in the 5hmC heatmap (Fig. 2a–d)].

It is well known that repression of gene expression by DNA methylation mainly occurs around the TSS [14]. We therefore characterized 5hmC distribution around TSS of annotated genes (Additional file 2: Fig. S2a–d) and found that the TSS of high expressed genes displayed higher levels of 5hmC than low expressed genes. When we increased the resolution of our analysis around the TSS (±5 kb), by considering the 5hmC signal every 50 bp (Additional file 2: Fig. S2e–h), we found that low expressed genes displayed a modest increase in 5hmC signal around the TSS, while highly expressed genes showed an overall higher signal characterized by a marked 150 bp deep around the TSS, surrounded by two 350-bp peaks (Additional file 2: Fig. S2e–h).

Enrichment of 5hmC in the body of highly expressed genes also correlated with active chromatin regions marked by H3K4me1, H3K27ac and H3K4me3 (Fig. 2a–d). In contrast, we observed no or an inverse correlation between 5hmC enrichment and regions containing repressive histone marks H3K27me3 and H3K9me3 (Fig. 2a–d). In all analyzed blood cell types, up to 40 and 25 % of the 5hmC were found within ChIP-seq peaks of active histone marks putative of poised enhancers H3K4me1 (40 %) or active enhancers H3K27ac (25 %) [15], while <7 % of 5hmC sites overlapped with repressive histone marks H3K27me3 and H3K9me3 (Additional file 2: Fig. S3). To rule out that the association of 5hmC with “active” histone modifications was attributed to an over-representation of CCGG sites within “active” histone ChIP-seq peaks, we compared the enrichment of 5hmC–CCGG to unmodified-CCGG and confirmed significant 5hmC enrichment in the vicinity of active histone marks (Additional file 2: Fig. S3). These results demonstrate that genes enriched with 5hmC are in an active chromatin state.

### In CD34+ cells, 5hmC associates with transcription factor binding at enhancers

**Fig. 3:**
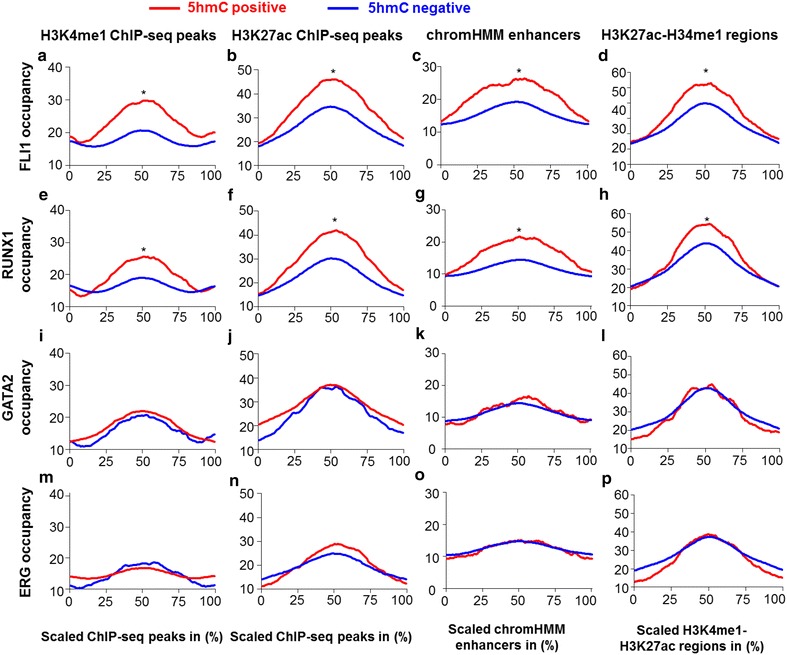
In BM-CD34+ cells, 5hmC associates with increased binding of FLI1 and RUNX1. Binding of FLI1 (**a**, **e**, **i**), RUNX1 (**b**, **f**, **j**), GATA2 (**c**, **g**, **h**) and ERG (**d**, **h**, **l**) at regulatory elements positive (*red line*) or negative (*blue line*) for 5hmC in BM-CD34+ cells. Regulatory regions were defined by chromatin enrichment with a specific histone modification or the ChromHMM, chromosome segmentation and Hidden Markov Model of Ernst and Kellis. Putative active enhancers were defined by H3K27ac ChIP-seq peaks or H3K4me1/H3K27ac ChIP-seq peaks. Poised enhancers were defined by H3K4me1 ChIP-seq peaks. A specific regulatory region was considered positive for 5hmC if it contained at least one hydroxymethylated cytosine. Since regulatory regions throughout the genome defined by ChIP-seq peaks or ChromHMM were of different lengths, we scaled regulatory regions to the same length (scaled ChIP-seq peaks, *x*-axis) and analyzed TF occupancies (*y*-axis; read coverage patterns). Differences between TF enrichment were assessed using the Kolmogorov–Smirnov test to quantify the distance between the empirical distribution function of the TF enrichment in regulatory regions positive versus negative for 5hmC, *significant differences in TF occupancy with *D* > 0.45 and *p* < 10^−10^

TF binding is central in the regulation of gene expression during hematopoiesis and is known to be affected by epigenetic modifications such as DNA methylation [16]. We therefore used publically available ChIP-seq data sets [17] and investigated whether the presence of 5hmC at gene regulatory regions could affect binding of FLI1, RUNX1, GATA2 and ERG, TFs crucial for blood development. We found that all four TFs bound chromatin enriched with H3K4me1, H3K27ac and H3K4me3 (Fig. 3; Additional file 2: Fig. S4), while no TF binding was detected in chromatin marked with H3K27me3 or H3K9me3 (Additional file 2: Fig. S4). At putative poised enhancers (H3K4me1 peaks), active enhancers (H3K27ac peaks or dually marked H3K27ac/H3K4me1 regions), as well as predicted enhancers using the Hidden Markov Model of Ernst and Kellis: ChromHMM [18], RUNX1 and FLI1 binding was enhanced in the presence of 5hmC (Fig. 3a–h). The presence of 5hmC did not influence GATA2 and ERG binding (Fig. 3i–p).

RRHP allows us to assess 5hmC distribution in a CCGG-specific context [11]. Since TF binding is sequence specific, we ensured that increased binding of FLI1 and RUNX1 at enhancers positive for 5hmC was not biased toward the CCGG sequence. We therefore compared TF binding at histone modification peaks positive for CCGG and 5hmC versus histone peaks positive for CCGG but negative for 5hmC and confirmed a significantly higher binding of FLI1 and RUNX1 at active enhancers enriched with 5hmC (Additional file 2: Fig. S5). Taken together, these results show that the presence of 5hmC in regulatory regions may increase the recruitment of key hematopoietic TFs such as RUNX1 and FLI1 at enhancers.

### 5hmC marks genes linked to hematopoietic differentiation

**Fig. 4:**
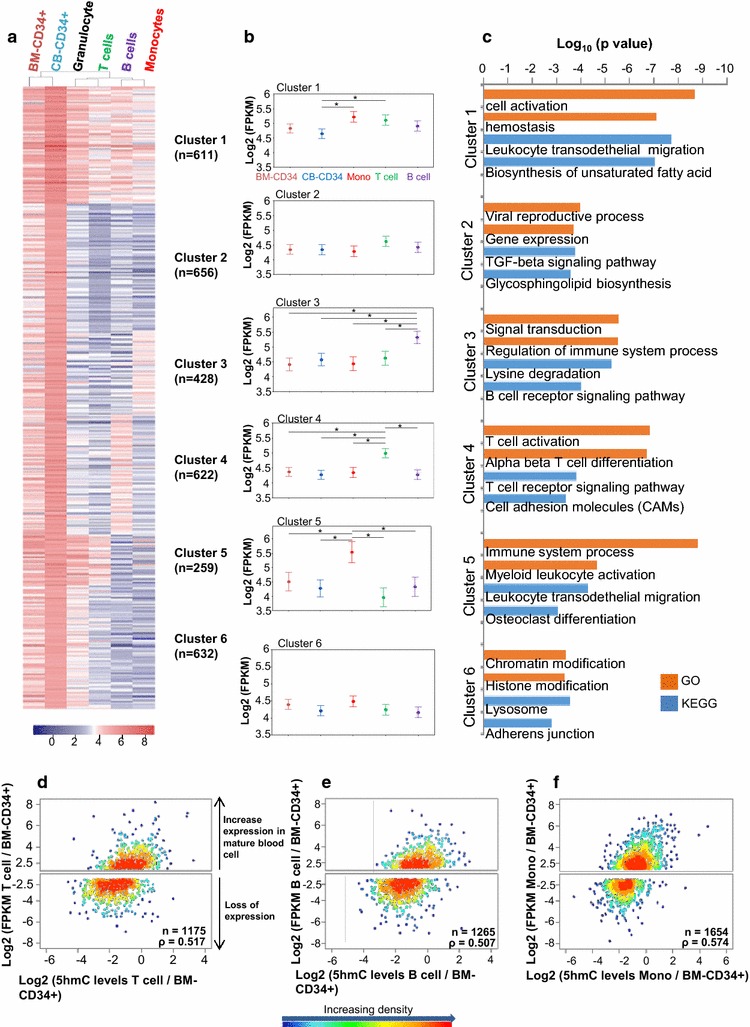
5hmC marks genes important for blood cell type function. **a** Using Euclidian cluster calling and the complete linkage method, 3208 genes (from cluster E in Additional file 2: Figure S4) were clustered according to their gene body 5hmC levels. The mean levels of gene body 5hmC from two biological replicates were used for granulocytes and CB-CD34+ cells. **b** Log2 of FPKM-averaged gene expression of all genes in the different clusters across the cell types. Only genes with FPKM >1 were included. Averaged log2 (FPKM) is shown ±95 % CI. *Two-tailed unpaired Student’s *t* test; *p* < 0.001. **c** Gene ontology (GO) and Kyoto Encyclopedia of Genes and Genomes (KEGG) analyses of genes falling in the six clusters. Only the first two GO and KEGG terms are listed. Heat density plots showing gene-wise changes in gene expression (FPKM, *y*-axis) versus changes in gene body 5hmC (*x*-axis) between progenitor BM-CD34+ cells and mature blood cells: T cells (**d**), B cells (**e**) and monocytes (**f**). Only genes with FPKM and gene body 5hmC >1 were plotted. The logarithmic fold change in gene expression needed to be ≥2 to define increase or loss of gene expression in mature blood cells. In each plot, the Spearman rank correlation coefficient *ρ* and the number (*n*) of genes plotted are shown (exact permutation test for testing the null hypothesis of no correlation, two-tailed, *p* < 5 × 10^−20^)

To investigate whether 5hmC marks genes linked to hematopoiesis in a cell-type-specific manner, we performed unsupervised clustering based on gene body 5hmC levels for all annotated genes. We identified 3208 genes, which contained high gene body 5hmC levels in all analyzed hematopoietic cell types (Additional file 2: Fig. S6a; cluster E). Gene set enrichment analysis using GO and KEGG confirmed that these genes strongly associated with blood cell function (Additional file 2: Fig. S6b). A second unsupervised cluster analysis subdivided the 3208 genes into six clusters (Fig. 4a; Additional file 3: Table S2), allowing us to identify groups of genes with differential gene body 5hmC levels across the mature blood cell types and pointing to specific 5hmC profiles associated with definite blood cell function. Averaged expression of genes found in each cluster indicated that genes maintaining high gene body 5hmC levels during differentiation of CD34+ cells into a specific mature blood cell type were highly expressed in the respective blood cell type (Fig. 4b) and recapitulated mature blood cell function (GO and KEGG analysis; Fig. 4c).

Such specific changes of intragenic 5hmC levels during CD34+ cell differentiation into a mature blood cell type led us to investigate how changes in gene body 5hmC levels correlated with changes in gene expression. We found that an increase in gene expression during differentiation of BM-CD34+ cells into a mature cell type associated with the preservation or gain of gene body 5hmC levels, while loss of gene expression in the committed cells correlated with a significant decrease in gene body 5hmC levels (Fig. 4d–f). Similar results were obtained when CB-CD34+ cells were used as reference cell type (Additional file 2: Fig. S7). Taken together, our results suggest that in CD34+ cells, 5hmC marks key genes required for lineage specification and mature blood cell function.

### Concomitant high gene body 5hmC levels and promoter DNA methylation prime genes for expression or repression during CD34+ cell differentiation

Our data indicated that in CD34+ cells, 5hmC primes genes for further expression in differentiated cells. To validate dynamic changes of cytosine modifications associated with the regulation of gene expression during hematopoiesis, key genes were selected from the clusters obtained in Fig. 4a. TNFRSF25 is a marker of activated T cells, CD19 a surface marker of mature B cells, while FOXO1 is a TF important for lymphoid lineage commitment. AZU1 has been selected as a regulator of myeloid cell function and RUNX1 as a TF important for CD34+ cell homeostasis. We measured gene expression, promoter DNA methylation and integrated it with gene body 5hmC levels.

**Fig. 5:**
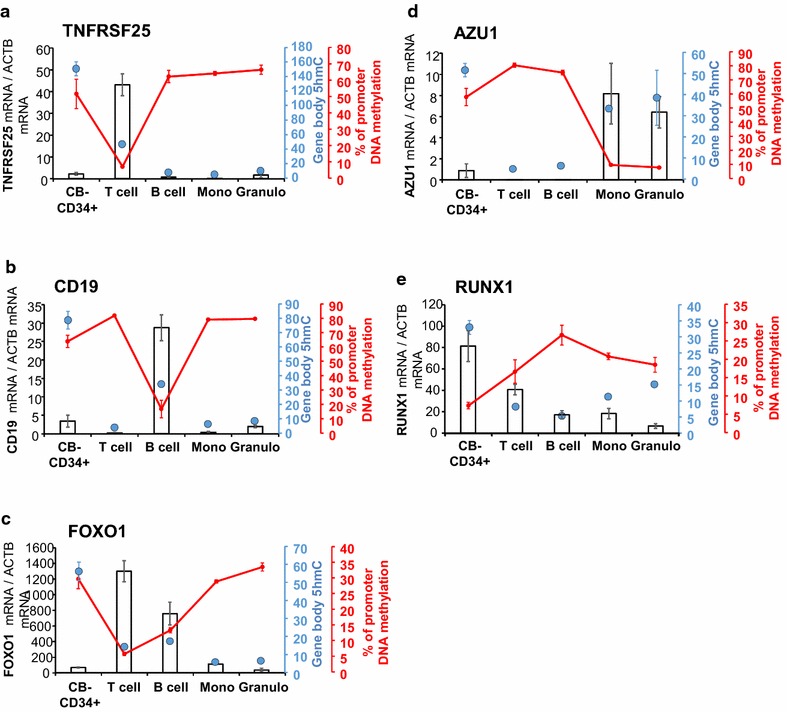
Cytosine modification (5hmC, 5mC) changes correlate with gene expression. Gene expression (*bars*), promoter DNA methylation (*red line*) and 5hmC gene body levels (*blue dots*) were assessed for *CD19* (**a**), *TNFRSF25* (**b**), *FOXO1* (**c**), *AZU1* (**d**) and *RUNX1* (**e**). Gene expression levels were measured by real-time quantitative reverse transcription PCR and normalized to beta-actin levels. Promoter DNA methylation was assessed by bisulfite conversion and pyrosequencing. The percentage of methylation represents the average methylation of CpG sites in the region, and gene body 5hmC levels were measured by RRHP

In T cells, B cells, monocytes and granulocytes, genes highly expressed contained significant levels of gene body 5hmC and low promoter DNA methylation. In contrast, high promoter DNA methylation and lack of gene body 5hmC were associated with low gene expression (Fig. 5). In CD34+ cells, DNA methylation of FOXO1, TNFRSF25, CD19 and AZU1 promoters was concomitant with high 5hmC gene body levels (Fig. 5a–d). RUNX1, which is higher expressed in CD34+ cells, displayed lower promoter methylation in these cells (Fig. 5e). Our data indicate that in CD34+ cells, genes containing 5hmC and 5mC are primed for active transcription in mature blood cells. During differentiation, loss of gene body 5hmC will result in gene repression, while loss of promoter DNA methylation will allow gene expression.

## Discussion

Here, we characterized for the first time the genome-wide distribution of 5hmC in human CD34+ progenitor and mature blood lineage cells. Our main findings are that (1) the presence of 5hmC in gene bodies positively correlates with gene expression and active chromatin state, (2) in CD34+ cells, 5hmC primes the expression of genes that are important for myeloid and lymphoid cell differentiation, and (3) in CD34+ cells, the presence of 5hmC at enhancers may enhance binding of key hematopoietic TFs.

We showed that cells with higher renewal potential present higher levels of 5hmC compared to differentiated blood cells. Importantly, differentiated blood cell types maintained substantial levels of 5hmC, suggesting a regulatory role for 5hmC rather than simply being an intermediate product during the demethylation process. Gene expression profiles of analyzed blood cells types revealed that highly expressed genes display higher levels of 5hmC in the immediate vicinity of TSS. This is in accordance with recent reports [10] and clearly demonstrates that 5hmC is found in active/open chromatin regions in hematopoietic cells.

Our study highlights the importance of dynamic changes of 5hmC distribution during CD34+ cell differentiation. A recent study by Madzo et al. [8] investigated the role of 5hmC during in vitro erythroid differentiation and also highlighted changes of 5hmC distribution during erythroid cell formation. Here, we used primary human cells to assess 5hmC function and distribution, which is of great importance since it has previously been shown that 5hmC levels decrease quickly during in vitro cell culture [19]. Two recent reports studied the role of 5hmC during mouse T and human B cell development into Th1/Th2 cells or plasma cells, respectively [9, 10]. Importantly, our study included multipotent CD34+ cells and mature blood cell types, which are still poorly studied with respect to 5hmC, and provides a valuable resource for a systematic analysis of 5hmC function during the whole hematopoietic differentiation process.

Several studies, including ours, link 5hmC to histone modifications indicative of enhancer [20]. In addition, we show that in CD34+ cells, the presence of 5hmC at putative active or poised enhancers associates with increased binding of RUNX1 and FLI1. Whether 5hmC is deposited passively in gene regulatory regions due to chromatin activity, or whether it functionally regulates TF binding and therefore gene expression remains incompletely understood. Madzo et al. [8] observed a dramatic decrease of 5hmC levels during erythroid differentiation compared to CD34+ cells, which is in agreement with our results. Moreover, the authors observed a strong correlation between loci that gained 5hmC and binding of TF known to be important for erythropoiesis, supporting that 5hmC may be a positive regulator of TF function. In agreement with our study, these results highlight that 5hmC may play an important role in regulating TF binding affinity to their target binding sites or in priming chromatin-remodeling processes to allow TFs to exert their functions. Therefore, inactivation of TET2, which is known to affect blood stem/progenitor cell renewal and differentiation [7, 21], would lead to reduced 5hmC levels and may consequently lead to aberrant TF binding and impaired gene expression.

Recent studies suggest that 5hmC has a destabilizing effect on DNA structure [22, 23], while 5mC stabilizes chromatin [24]. In CD34+ cells, several genes showed concomitant promoter DNA methylation and high gene body 5hmC levels. This “bivalent” 5mC/5hmC status may prime genes for further epigenetic regulation of their expression during hematopoietic development. Loss of gene body 5hmC would result in gene repression, while loss of promoter DNA methylation would allow expression. The coexistence of epigenetic marks with opposite effects on gene expression has previously been described for histone marks (H3K4me3 and H3K27me3). The presence of “bivalent histone domains” maps key developmental genes that need to be turned on or off during differentiation [25]. Importantly, 5hmC was shown to associate with “bivalent histone domains” in ESC [26]. Our analysis suggests that in CD34+ cells, gene body 5hmC marks genes whose expression can be epigenetically regulated during differentiation into a specific mature blood cell type. Taking our results in perspective to those of others, we find that in multipotent CD34+ cells, 5hmC maps genes important for lineage specification and suggest that 5hmC may directly influence cell fate decisions.

## Conclusions

We used a single-base resolution method to show that during human hematopoiesis, 5hmC accompanies extensive chromatin reprogramming, including TF binding, and marks cell-specific genes actively transcribed in differentiated cells that are known to be important for myeloid and lymphoid lineage commitment.

## Methods

### Human blood cells

Umbilical cord blood was collected by sterile procedure from healthy pregnancies. Peripheral blood was obtained from adult healthy donors. All subjects provided written informed consent. Blood cells were separated by density gradient, followed by cell-type-specific isolation using MACS MicroBeads (Miltenyi, Biotec). Bone marrow CD34+ cells, from a unique donor, were purchased from StemCell Technologies. Granulocytes, CD4+, CD19+, CD14+ and BM-CD34+ cells were obtained from males and females between 30 and 50 years old. All experiments were approved by the Regional Committee for Medical and Health Research Ethics (2012/588/REK and 2012/1969/REK).

### Reduced representation of hydroxymethylcytosine profiling (RRHP)

RRHP was performed as previously described [11] by Zymo Research on service basis (Zymo Research, California, USA). In brief, genomic DNA was fragmented using MspI, the sticky ends obtained were reconstituted using specific adapters, 5hmC was then glucosylated, the modified fragments were submitted to a second MspI digestion, and the CCGG sites with a glucosylated 5hmC were protected from digestion and further amplified and sequenced using next-generation sequencing. For sequencing, equal volumes of each amplified library were pooled and diluted to 8 pM. Samples were six-plexed into a single 50-bp sequencing using an Illumina HiSeq. FASTQ files were aligned to the human reference genome (hg19) with Bowtie 0.12.8 using default parameters and best. Only mapped sequencing reads beginning with a CCGG tag were considered in the analysis. The number of sequencing reads at a CCGG site was considered to be proportional to the level of hydroxymethylation. To compare positive 5hmC among the different cell types we used a more stringent read cutoff than in Petterson et al. [11]. A CCGG site was considered hydroxymethylated if the number of sequencing reads at this site was >101.8 reads, which is the mean read count + SEM for all CCGG sites in hESC (the cell type with highest 5hmC levels). Information regarding the RRHP data for each sample and replicates can be found in Additional file 1: Table S1.

To compare the positive 5hmC within the different cell types we use a more stringent read cutoff than the original method paper [11]. Based on our more stringent selection of 5hmC sites we found a strong correlation in replicated cell types (hESC, CB-CD34+ and granulocytes) with Pearson’s correlation *r* > 0.75 and *p* < 0.05 (Additional file 1: Table S1).

Gene body 5hmC levels were determined by dividing the number of sequencing read at CCGG sites by the number of CCGG sites in the gene body.

### DNA isolation and LC/MS/MS analysis

DNA from hESC (*n* = 1), CB-CD34+ (*n* = 2), as well as CD4+ T cells (*n* = 3), CD19+ B cells (*n* = 1), CD14+ monocytes (*n* = 3) and granulocytes (*n* = 3), was isolated after proteinase K (Sigma, St. Louis, MO, USA) and RNase A digestion (Qiagen, Germany) followed by phenol–chloroform extraction and ethanol precipitation. For LC/MS/MS, DNA was hydrolyzed to nucleosides by incubation with nuclease P1, snake venom phosphodiesterase and alkaline phosphatase (Sigma-Aldrich, St. Louis, MO, USA). Three volumes of methanol were added after digestion, and the reactions were centrifuged at 16,000*g* for 30 min. The supernatants were dried under vacuum, and resulting residues were dissolved in 50 µl 5 % (v/v) methanol for analysis. Chromatographic separation of nucleosides was performed using a Shimadzu Prominence HPLC system with a Ascentis Express C18 150 × 2.1 mm i.d. (2.7 µm) reverse-phase column equipped with an Ascentis Express C18 12.5 × 2.1 mm i.d. (2.7 µm) guard column (Agilent Technologies, USA), with a flow rate of 0.2 ml/min at ambient temperature. The mobile phase consisted of A (0.1 % formic acid in water) and B (0.1 % formic acid in methanol), starting with 95 % A/5 % B for 0.5 min, followed by a 6.5-min linear gradient of 5–50 % B, 2 min with 50 % B and 6-min re-equilibration with the initial mobile phase conditions. Online mass spectrometry detection was performed using an AB Sciex 5000 triple quadrupole mass spectrometer (AB Sciex, USA) with TurboionSpray probe operating in positive electrospray ionization mode. The deoxyribonucleosides were monitored by multiple reaction using the mass transitions 228.1 → 112.1 (dC), 242.1 → 126.1 [5-me(dC)] and 258.1 → 142.1 [5-hm(dC)].

### RNA sequencing

RNA from CB-CD34+, CD4+ T cells, CD14+ monocytes and CD19+ B cells was purified using the RNeasy Mini Kit (Qiagen). Samples with an RNA integrity number value >8 were used for sequencing. Libraries were prepared using TruSeq RNA Sample Preparation Kit v2 (Illumina) following the manufacturer’s instructions and sequenced with 100-bp paired-end reads on a HiSeq2000 Illumina. RNA-Seq data sets of BM-CD34+ cells (GSE63569) were downloaded from the gene expression database of Gene Expression Omnibus (GEO) [27]. Reads were aligned to the human reference genome (hg19) by TopHat v2.0.13 [28] and assembled by cufflinks v2.1.1 [28] using default parameters. Quantification of gene expression was performed using cufflinks v2.1.1 with default parameters to obtain fragment per kilobase million (FPKM) value.

### Bisulfite conversion and pyrosequencing

DNA bisulfite conversion and pyrosequencing were performed as previously described [29]. Primers used for pyrosequencing, target sequences analyzed and number of CpGs assessed are listed in Additional file 4: Table S3.

### Analysis of publically available chromatin immunoprecipitation sequencing (ChIP-seq)

Histone modification and TF-ChIP-seq data sets were publicly available. The accession numbers and description of histone modification ChIP-seq data sets are listed in Additional file 5: Table S4. FLI1, RUNX1, GATA2 and ERG TF-ChIP-seq data sets [17] were downloaded from the gene expression database of GEO (GSE45144). Raw reads were aligned to the human reference genome (hg19) using Novoalign v2.08.02 (http://www.novocraft.com/products/novoalign/) and default parameters. Reads with low quality ≤20 were removed. Chromatin enriched with a specific histone modification or TF binding was identified using MACS v1.4.1 with default parameters and respective input samples as background.

### Prediction of active and poised enhancers

Using histone modification ChIP-seq peaks we defined active enhancers as regions enriched with H3K27ac, while putative poised enhancers were regions enriched with H3K4me1. We also used regions enriched with both histone marks (H3K27ac and H3K4me1); here, an H3K27ac peak was considered to be positive for H3K4me1, if at least 25 % of the H3K27ac peak was covered by H3K4me1 peaks. The H3K27ac/H3K4me1 regions were defined as active enhancers if they lacked TSS (TSS ±1000 bp). We also used the predicted enhancer region of CD34+ cells using the ChromHMM segmentation of Ernst and Kellis [18]. Predicted enhancer regions using the Hidden Markov Model were obtained from The NIH Roadmap Epigenomics Mapping Consortium.

### Quantitative RT-PCR

cDNA synthesis and qRT-PCR were performed as previously described [29]. Expression levels of target genes were normalized to beta-actin expression. Primer sequences are listed in Additional file 6: Table S5.

### Statistical analyses

Student’s unpaired two-tailed t test was used to compare means. In case of non-normal distribution, the Mann–Whitney test was used to compare medians. *p* value <0.05 was considered statistically significant. Hierarchical clustering was applied to log2 gene body 5hmC levels using the Euclidean distance and complete linkage method. We used HOMER to perform GO and KEGG functional enrichment analyses, based on all RefSeq genes as reference. The deepTools suite [30] and the computeMatrix command in scale-region mode were used to extract the scores of enrichment and produce the heatmaps of 5hmC and histone modification enrichments in gene bodies. 5hmC enrichment in gene bodies was obtained by normalizing RRHP-CCGG reads to distribution of CCGG sites in the human genome. Histone modification enrichment was obtained by normalizing ChIP-seq reads to input reads. The deepTools suite was used to analyze TFs occupancies (*y*-axis) in regulatory regions (scaled ChIP-seq peaks, *x*-axis) by normalizing ChIP-seq reads for RUNX1, FLI1, GATA2 or ERG to input reads. Kolmogorov–Smirnov test was used to quantify the distance between the empirical distribution function of two conditions.

### Availability of data and materials

The data sets supporting the conclusions of this article are available in the NCBI GEO (http://www.ncbi.nlm.nih.gov/geo/) under accession number GSE69905.

## Additional files

10.1186/s13072-016-0070-8 Summary of RRHP sequencing data. Table describes RRHP sequencing depth, percentage of aligned reads, raw and normalized 5hmC counts as well as correlation.
10.1186/s13072-016-0070-8 Quantification of 5-methylcytosine using LC/MS/MS. Quantification of 5-methylcytosine (5mC) per 10^6^ unmodified bases in hESC (n = 1), CB-CD34+ (n = 2), CD4+ T cells (n = 3), CD19+ B cells (n = 1), CD14+ monocytes (n = 3), and granulocytes (n = 3) assessed by LC/MS/MS. n indicates the number of independent DNA measured for each cell type. **Figure S2**: 5hmC at transcription start sites. Average enrichment of 5hmC RRHP reads (signal) along a 100 kb window (**a-d**) and 5 kb window (**e–h**) around the transcription start sites (TSS) of high and low expressed genes in the indicated hematopoietic cell types. Mean gene FPKM was used as threshold to dichotomize gene expression in high/low according to RNA-seq data in the same cell-types. **Figure S3**: 5hmC and histone modifications. Percentage of 5hmC or CCGG sites encompassed in ChIP-seq peaks of indicated histone modifications. The mean percentage of 5hmC in ChIP-seq peaks from two independent RRHP measurements in CB-CD34+ cells and one in BM-CD34+ are shown ± SEM. The mean percentages of CCGG sites found in ChIP-seq peaks of CD34, T cell, monocyte and B cell are shown.*** or * indicate significant difference between CCGG distribution in ChIP-seq peaks *versus* 5hmC distribution in ChIP-seq peaks of hematopoietic cells. *** p < 0.0001, * p < 0.05; Student’s unpaired two tailed t-test. **Figure S4**: 5hmC and transcription factor occupancy at inactive chromatin. FLI1, RUNX1, GATA2 and ERG occupancies at putative active promoters (H3K4me3 peaks), putative inactive chromatin enriched with H3K27me3 or H3K9me3. A specific ChIP-seq peak was considered positive for 5hmC when it contained at least one hydroxymethylated cytosine. In histone modification ChIP-seq-peaks positive or negative for 5hmC we assessed how TF-ChIP-seq reads were distributed which reflected the level of occupancy of an inactive chromatin region by a TF. The diagrams show the occupancy of a TF in inactive chromatin positive (red line) or negative (blue line) for 5hmC. **Figure S5**: Transcription factor occupancy at putative regulatory elements. FLI1, RUNX1, GATA2, ERG occupancies at regulatory elements in BM-CD34+ cells. Regulatory regions were defined by chromatin enrichment with histone modifications: H3K4me1 or H3K27ac-ChIP-seq peaks defined putative active enhancers, H3K4me3-ChIP-seq peaks defined active promoter regions. A specific regulatory region was considered positive for 5hmC if it contained at least one 5hmC site. In histone modification ChIP-seq-peaks we assessed how particular transcription factor ChIP-seq reads were distributed, which is proportional to binding. Occupancy of the indicated transcription factors in regulatory elements containing 5hmC-positive CCGG (red line) or 5hmC-negative CCGG (blue line) sites are shown. Enrichment of TFs (y-axis) at regulatory regions (scaled ChIP-seq peaks, x-axis). Differences between TF enrichment were assessed using the Kolmogorov–Smirnov test to quantify the distance between the empirical distribution function of the TF enrichment in regulatory regions positive *versus* negative for 5hmC, * indicates significant differences in TF occupancy with D > 0.3 and p < 5.10^-4^. **Figure S6**: Changes in 5hmC gene body levels. **a**: Heat map representation of all known human genes clustered according to their gene body 5hmC levels. Euclidian cluster calling was used to produce the heat map. The five clusters identified are labeled with a letter and the number of genes *per* cluster is indicated. The mean levels of gene body 5hmC from two biological replicates were used for granulocytes and CB-CD34+ cells. **b:** Gene ontology (GO) and Kyoto Encyclopedia of Genes and Genomes (KEGG) analyses of genes identified in the five clusters in panel A. The first two GO and KEGG terms are listed. **Figure S7**: Changes in 5hmC gene body levels versus changes in expression. Heat density plots depict the changes in gene expression (FPKM, *y* axis) *versus* changes in gene body 5hmC (*x* axis) from progenitor cells (CB-CD34+ cells) to mature blood cells: T cells **(a)**, B cells **(b)** and monocytes **(c)**. Only genes with FPKM and gene body 5hmC > 1 were plotted. The logarithmic fold change in gene expression needed to be > 2 to define gain or < 2 to define loss of gene expression. In each plot, the Spearman rank correlation coefficient ρ and the number (n) of genes plotted are shown (exact permutation test for testing the null hypothesis of no correlation, two tailed, p < 5 × 10^−20^).
10.1186/s13072-016-0070-8 Gene list described in Fig. 4a.
10.1186/s13072-016-0070-8 Primer used in pyrosequencing and coordinates of DNA sequence analyzed.
10.1186/s13072-016-0070-8 Publically available histone ChIP-seq data sets used.
10.1186/s13072-016-0070-8 Primers used in qRT-PCR.

## Abbreviations

5mC: 5-methylcytosine
5hmC: 5-hydroxymethylcytosine
BM: bone marrow
CB: cord blood
CD: cluster of differentiation
ERG: ETS-related gene
FLI1: friend leukemia integration 1
FPKM: fragment per kilobase million
GO: gene ontology
hESC: human embryonic stem cells
KEGG: Kyoto Encyclopedia of Genes and Genomes
RRHP: reduced representation of 5-hydroxymethylcytosine profiling
RUNX1: runt-related transcription factor 1
TET: ten-eleven-translocation
TF: transcription factor
TSS: transcription start site
TTS: transcription termination site
