# Supplementary material for: Changes of 5-hydroxymethylcytosine distribution during myeloid and lymphoid differentiation of CD34+ cells
Source: Epigenetics Chromatin. 2016 May 31;9:21. doi: 10.1186/s13072-016-0070-8 (PMC4888655; doi:10.1186/s13072-016-0070-8)
Supplement: Supplementary file 1 — 10.1186/s13072-016-0070-8 Summary of RRHP sequencing data. Table describes RRHP sequencing depth, percentage of aligned reads, raw and normalized 5hmC counts as well as correlation. [file 13072_2016_70_MOESM1_ESM.pdf]

Table S1: Summary of RRHP sequencing data

Sample ID	Cell type	Total Read Number	Aligned reads	All 5hmC+ CpG sites	5hmC+ CpG sites with more than 5 reads	normalized 5hmC+ CpG sites	Pearson correlation (r) with duplicate sample and p-value (p)
RRHP_hESC_38	hESC_1	44,675,164	89 %	2,073,058	960,965	64,653	r = 0.903 ; p < 5x10^-15
RRHP_hESC_22	hESC_2	51,034,136	81 %	1,959,479	1,067,214	83,132	
RRHP_CB_CD34_348	CB_CD34+ _1	43,009,150	92 %	1,857,698	890,018	45,456	r = 0.877 ; p < 5x10^-15
RRHP_CB_CD34_350	CB_CD34+ _2	42,363,277	91 %	1,843,420	890,484	43,511	
RRHP_BM_CD34	BM_CD34+	30,689,275	90 %	1,585,447	612,344	19,840	N/A
RRHP_Granulocytes_1	Granulocytes_1	41,182,246	90 %	1,789,541	799,185	13,2495	r = 0.771 ; p < 5x10^-15
RRHP_Granulocytes_2	Granulocytes_2	14,882,785	92 %	1,614,874	567,233	13,4005	
RRHP_CD4	CD4+	8,452,910	92 %	1,338,338	313,614	7,375	N/A
RRHP_CD14	CD14+	8,909,659	93 %	1,402,823	353,686	10,817	N/A
RRHP_CD19	CD19+	8,307,375	87 %	1,459,790	352,372	7,441	N/A
