# Supplementary figures and images for: Changes of 5-hydroxymethylcytosine distribution during myeloid and lymphoid differentiation of CD34+ cells
Source: Epigenetics Chromatin. 2016 May 31;9:21. doi: 10.1186/s13072-016-0070-8 (PMC4888655; doi:10.1186/s13072-016-0070-8)

Figure S1

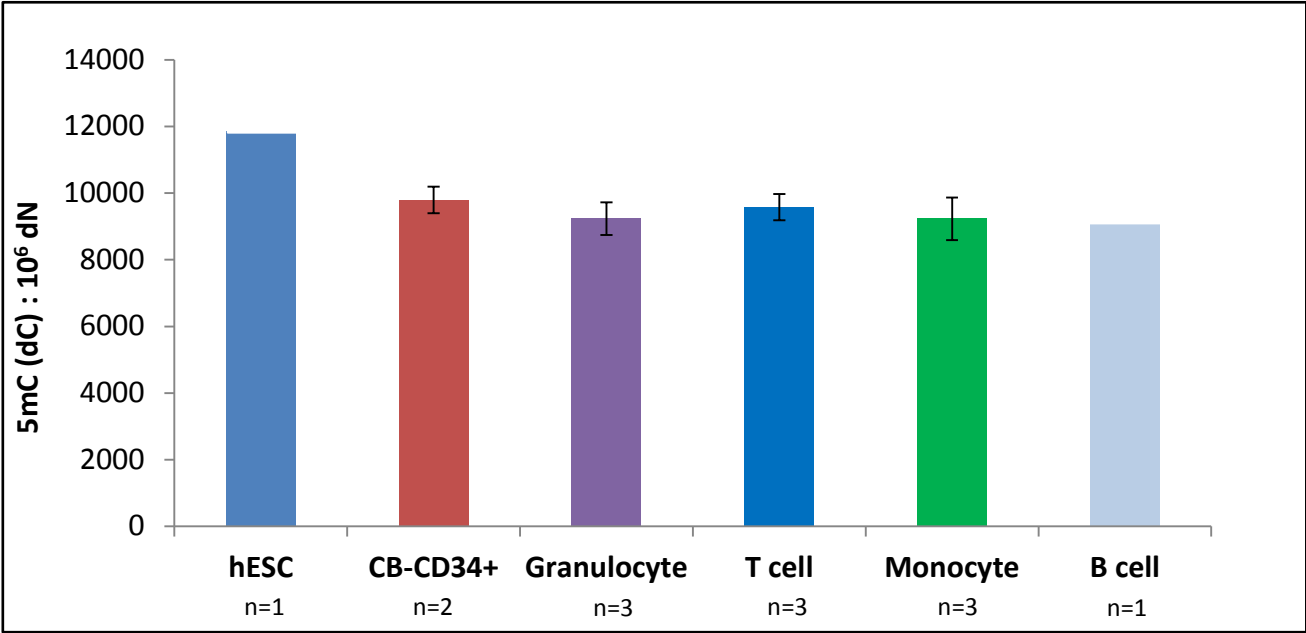

Figure S2

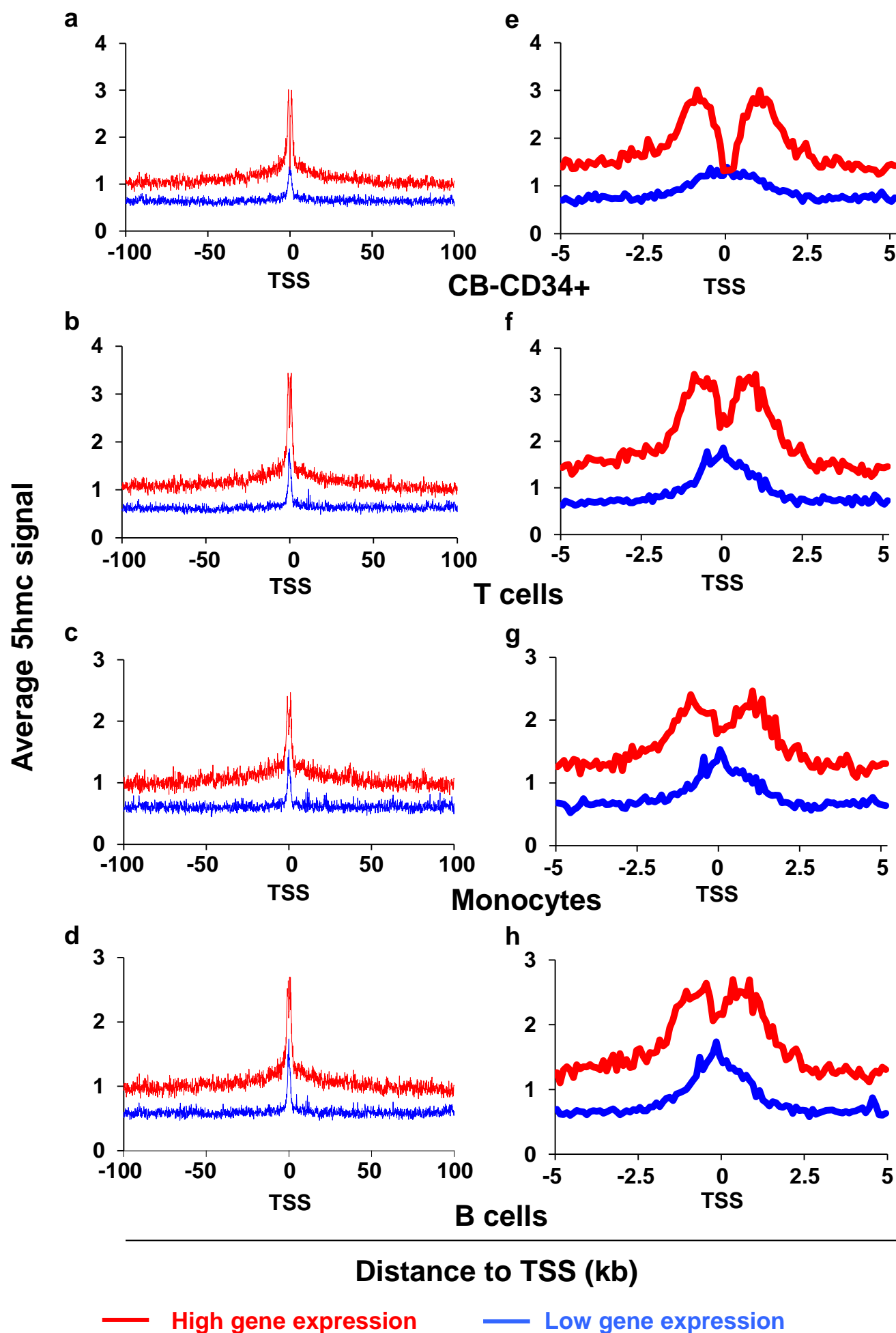

**Figure S3**

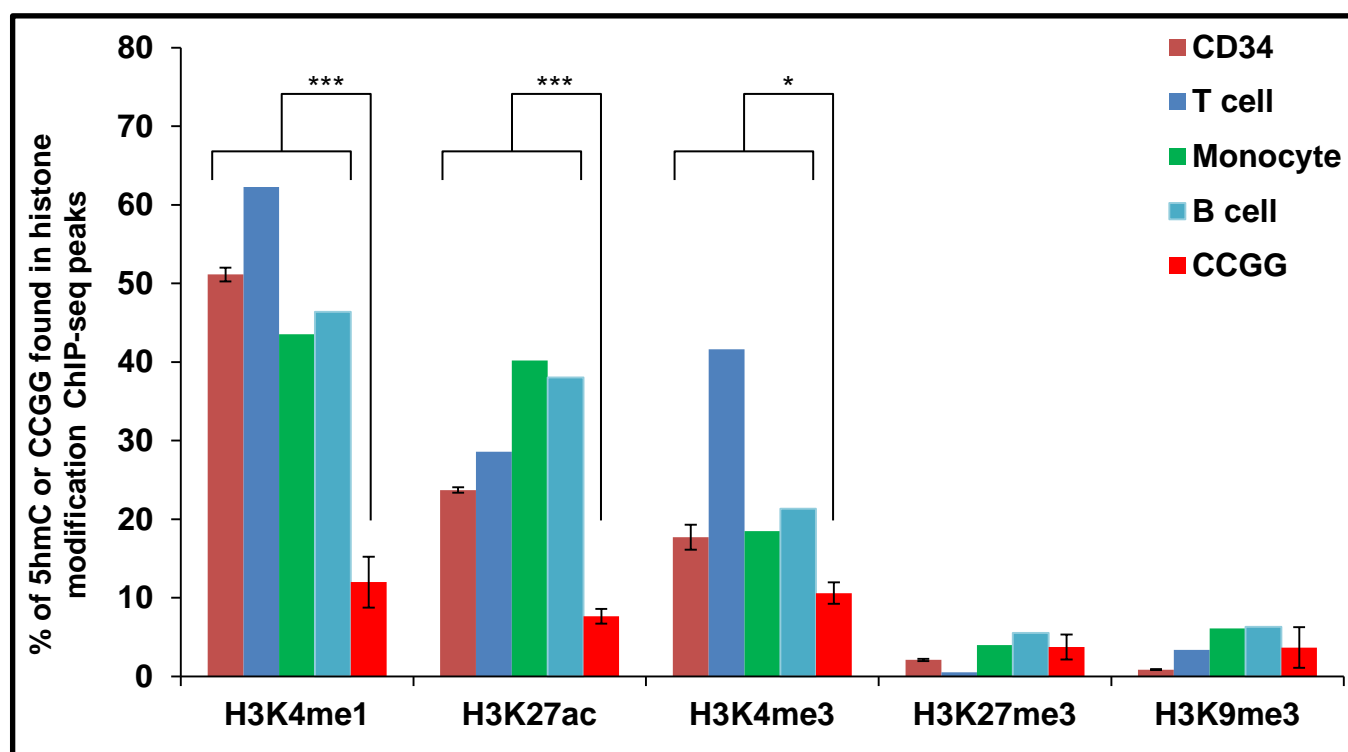

Figure S4

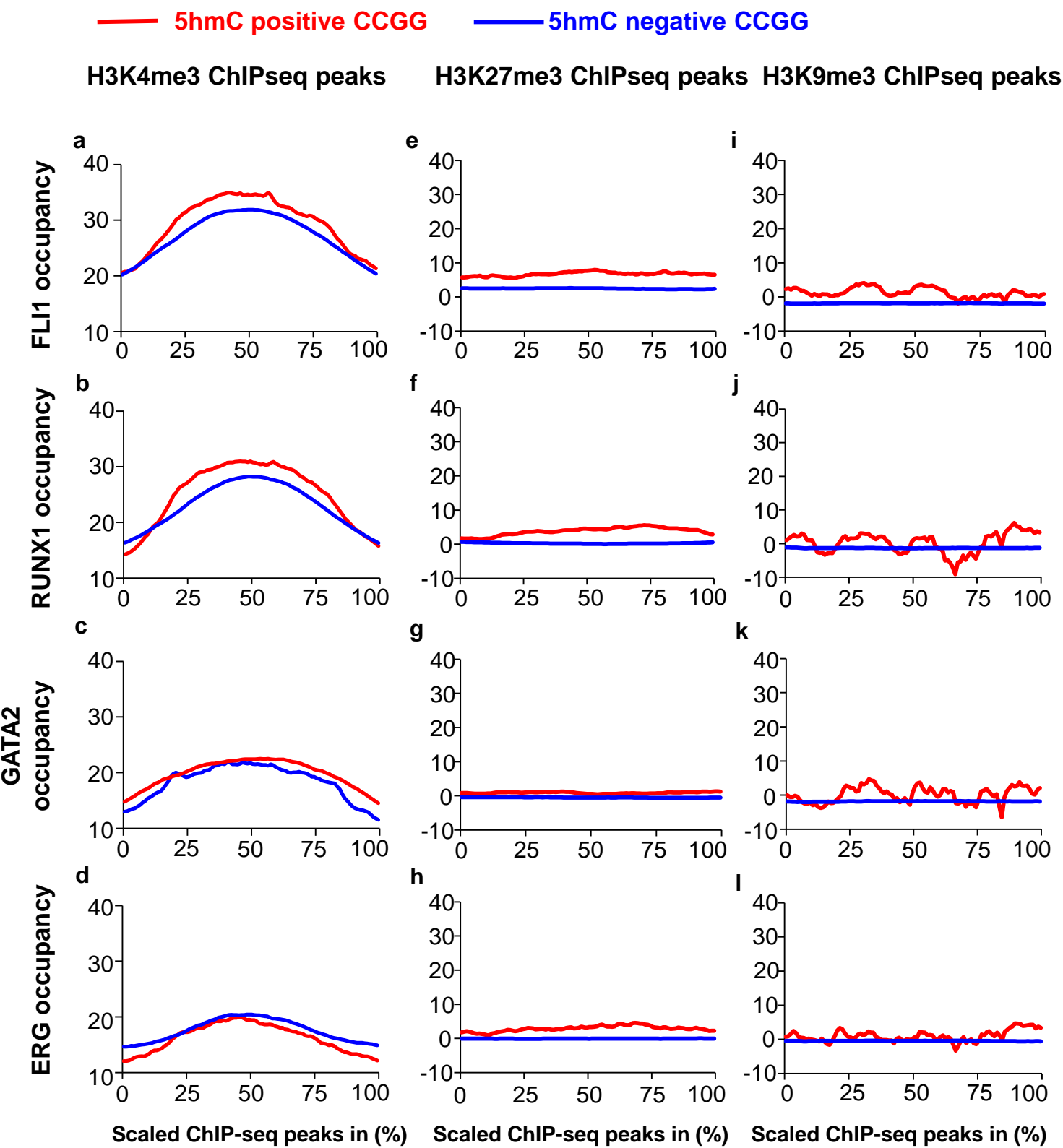

Figure S5

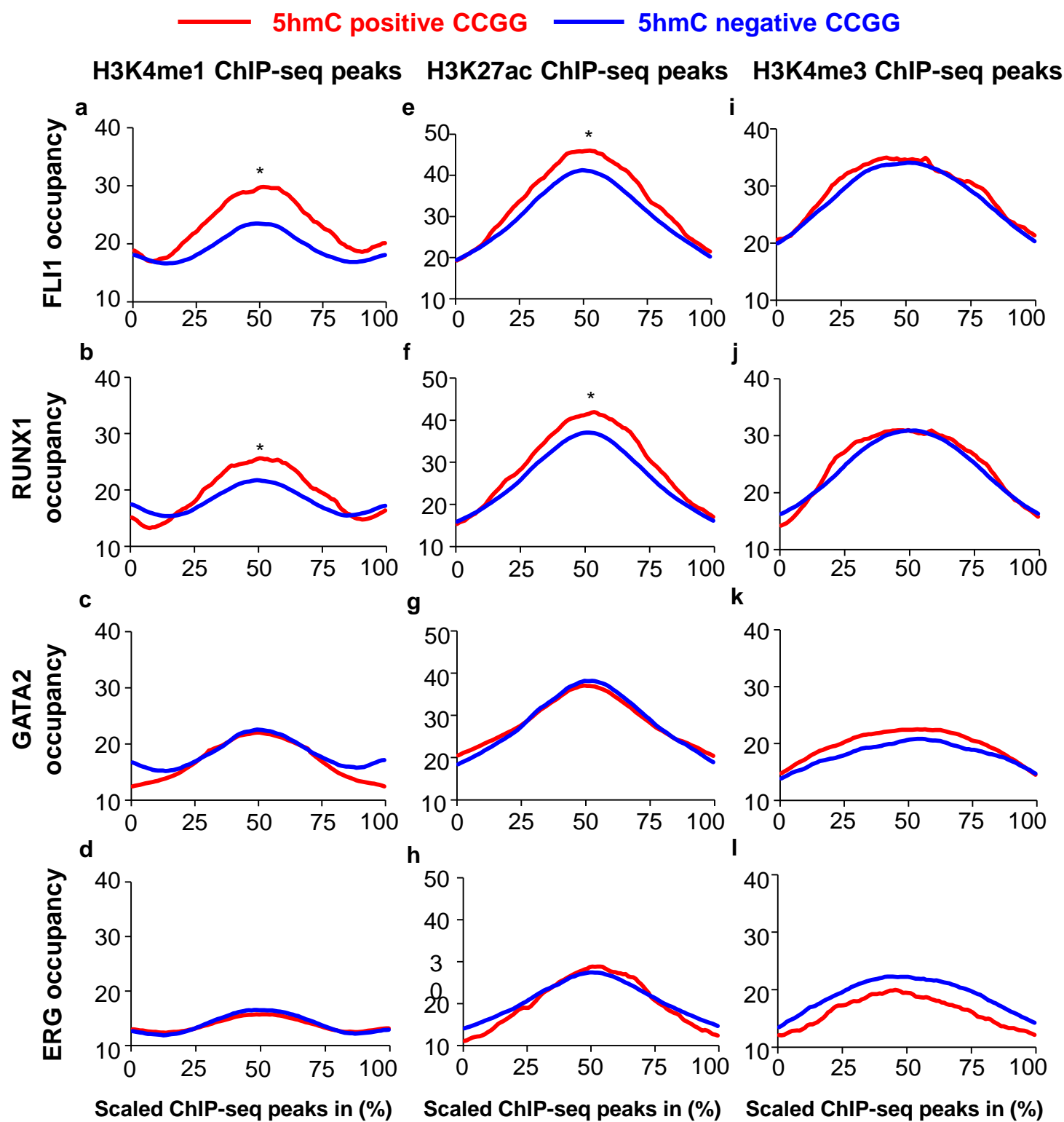

Figure S6

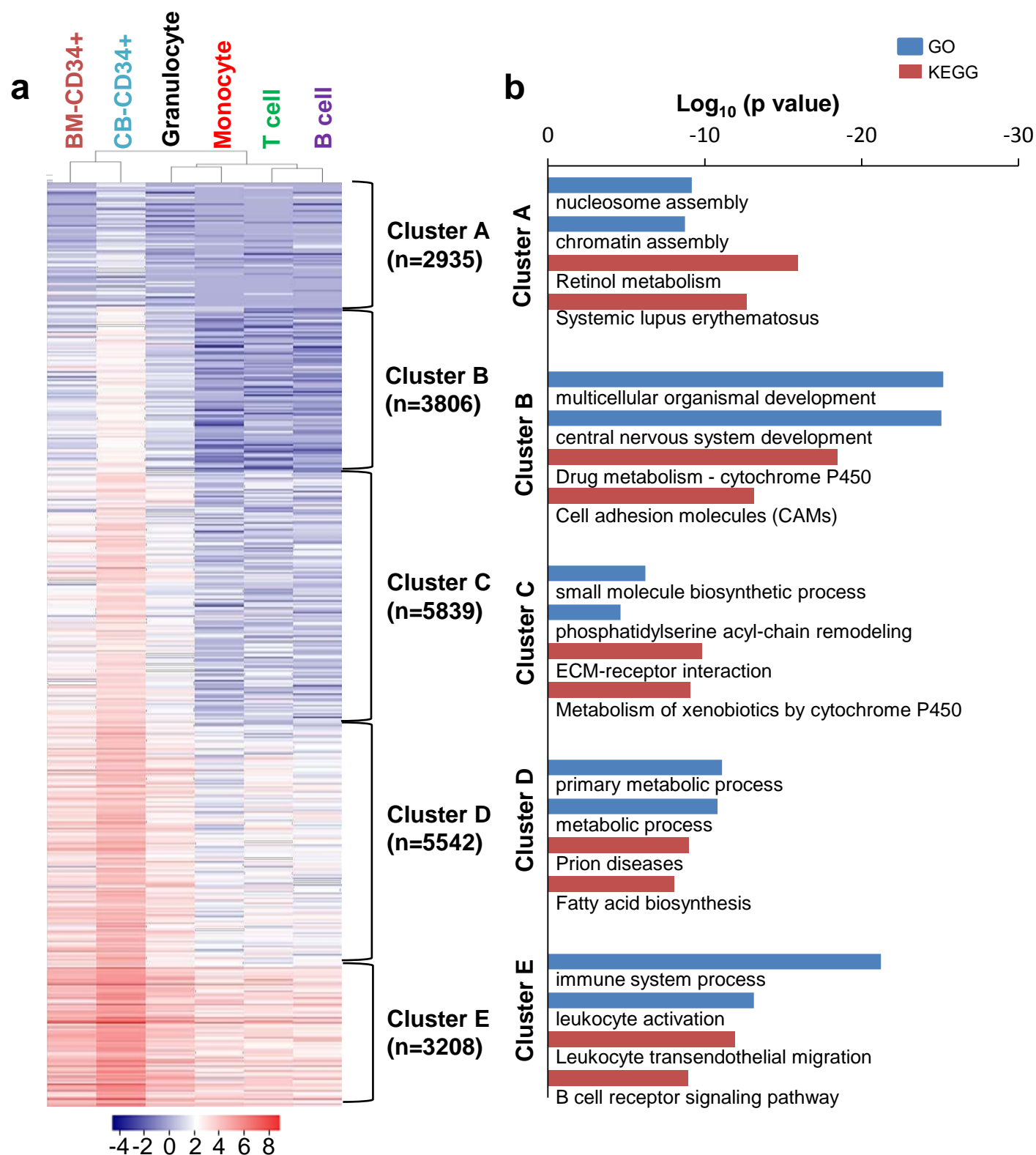

Figure S7

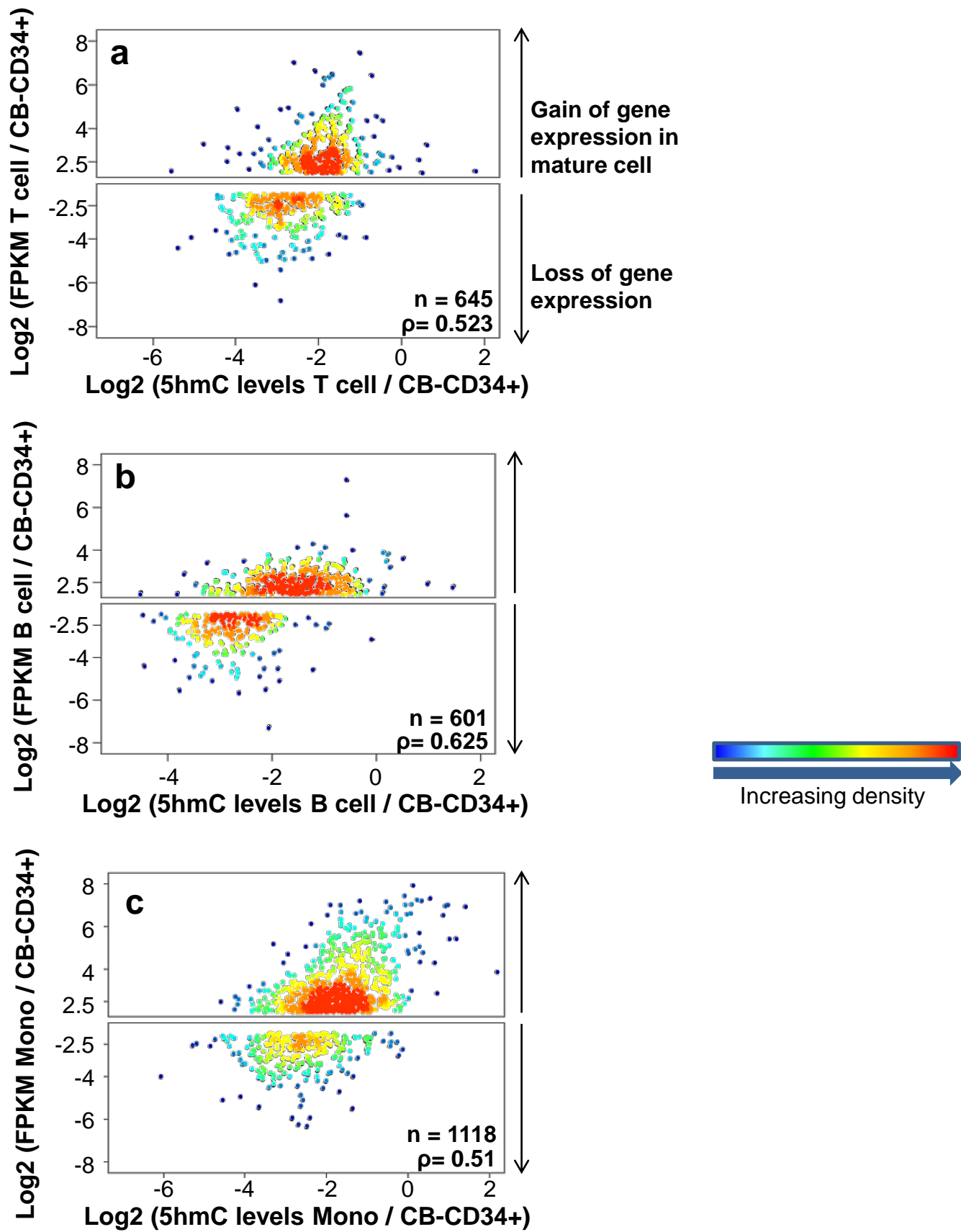

Supplement: Supplementary file 2 — 10.1186/s13072-016-0070-8 Quantification of 5-methylcytosine using LC/MS/MS. Quantification of 5-methylcytosine (5mC) per 106 unmodified bases in hESC (n = 1), CB-CD34+ (n = 2), CD4+ T cells (n = 3), CD19+ B cells (n = 1), CD14+ monocytes (n = 3), and granulocytes (n = 3) assessed by LC/MS/MS. n indicates the number of independent DNA measured for each cell type. Figure S2: 5hmC at transcription start sites. Average enrichment of 5hmC RRHP reads (signal) along a 100 kb window (a-d) and 5 kb window (e–h) around the transcription start sites (TSS) of high and low expressed genes in the indicated hematopoietic cell types. Mean gene FPKM was used as threshold to dichotomize gene expression in high/low according to RNA-seq data in the same cell-types. Figure S3: 5hmC and histone modifications. Percentage of 5hmC or CCGG sites encompassed in ChIP-seq peaks of indicated histone modifications. The mean percentage of 5hmC in ChIP-seq peaks from two independent RRHP measurements in CB-CD34+ cells and one in BM-CD34+ are shown ± SEM. The mean percentages of CCGG sites found in ChIP-seq peaks of CD34, T cell, monocyte and B cell are shown.*** or * indicate significant difference between CCGG distribution in ChIP-seq peaks versus 5hmC distribution in ChIP-seq peaks of hematopoietic cells. *** p < 0.0001, * p < 0.05; Student’s unpaired two tailed t-test. Figure S4: 5hmC and transcription factor occupancy at inactive chromatin. FLI1, RUNX1, GATA2 and ERG occupancies at putative active promoters (H3K4me3 peaks), putative inactive chromatin enriched with H3K27me3 or H3K9me3. A specific ChIP-seq peak was considered positive for 5hmC when it contained at least one hydroxymethylated cytosine. In histone modification ChIP-seq-peaks positive or negative for 5hmC we assessed how TF-ChIP-seq reads were distributed which reflected the level of occupancy of an inactive chromatin region by a TF. The diagrams show the occupancy of a TF in inactive chromatin positive (red line) or negative (blu [file 13072_2016_70_MOESM2_ESM.pdf]
