# Supplementary material for: Changes of 5-hydroxymethylcytosine distribution during myeloid and lymphoid differentiation of CD34+ cells
Source: Epigenetics Chromatin. 2016 May 31;9:21. doi: 10.1186/s13072-016-0070-8 (PMC4888655; doi:10.1186/s13072-016-0070-8)
Supplement: Supplementary file 3 — 10.1186/s13072-016-0070-8 Gene list described in Fig. 4a. [file 13072_2016_70_MOESM3_ESM.pdf]

Table S2: Gene list described in Figure 4a

Cluster1 (n=611)	Cluster2 (n=656)	Cluster3 (n=428)	Cluster4 (n=622)	Cluster5 (n=259)	Cluster6 (n=632)
SKI	PANK4	TNFRSF18	ISG15	S100A9	RNF223
LOC115110	KLHL21	AGMAT	TNFRSF4	SEMA4A	SLC2A7
PIK3CD	CA6	RCAN3	MIB2	FCGR2A	MTHFR
MIR4420	EPHA2	ZNF683	C1orf86	MIR1231	SPEN
DCLRE1B	ZDHHC18	LCK	SMIM1	MIR320B2	ARID1A
S100A6	SSBP3	S1PR1	TNFRSF25	TMEM72	THEMIS2
PMF1-BGLAP	THEM4	S100A2	FBXO2	RASSF4	RCC1
AIM2	S100A3	TTC24	RUNX3	GIF	B4GALT2
GAS5-AS1	ISG20L2	TRAF5	SYTL1	KCNE3	C1orf228
PTPRC	GJC2	IL2RA	MAP3K6	KLRK1	NASP
TRAF3IP3	PPIAP30	CASP12	FAM229A	IRAK3	RPF1
MIR3620	SH3PXD2A-AS1	ETS1	ELOVL1	HCAR2	CAPZA1
KLF6	RGS10	FKBP4	TCTEX1D4	HCAR3	POGZ
C10orf105	BNIP3	CD27-AS1	AMIGO1	LINC00462	S100A13
C10orf54	B4GALNT4	DGKA	C1orf56	COL4A2-AS1	C1orf85
MIR146B	MIR4298	DTX1	S100A10	MIR4706	MEF2D
ITPRIP	TNNT3	RASAL1	ATP8B2	TNFAIP2	ADAMTS4
FAM53B	MICAL2	HIP1R	ADAM15	ITGAX	RCSD1
ADAM8	MIR130A	LINC00426	CD247	C17orf105	SFT2D2
IFITM2	GANAB	SETDB2	ELK4	MIR3186	GPR137B
IFITM3	RCOR2	REM2	TMEM63A	LRG1	ALOX5
CTSD	TBX10	TTC9	ITPKB	RETN	NRBF2
TNNI2	B4GALNT3	MIR345	C1orf145	MIR4748	ZRANB1
LSP1	C1RL	BTBD6	TRIM17	LRRC25	INPP5A
C11orf21	SLC48A1	PRC1-AS1	LOC100499489	FFAR3	MIR202
FERMT3	COQ10A	DEXI	OTUD1	CEACAM4	RNH1
SF1	CDK4	MIR365A	PPIF	PRG1	CDHR5
EHD1	HCAR1	MIR138-2	CALHM2	TCF23	SYT8
ADRBK1	CHFR	DBNDD1	DUSP5	BCL11A	CARS
PPP1CA	FITM1	CLDN7	IFITM5	IL1RN	CELF1
TBC1D10C	PSME2	UBB	IFITM1	ZEB2	PTPRJ
RPS6KB2	PLEKHG3	CISD3	MIR210	LINC00656	AHNAK
CORO1B	TRMT61A	ZPBP2	LOC143666	VPREB1	OTUB1
ATG16L2	LINC00226	LRRC3C	CD6	CAMP	RIN1
MIR3649	WHAMMP2	TBX21	C11orf48	C3orf22	TCIRG1
PTPN6	MEX3B	SNORD104	ZBTB3	HTRA3	TPCN2
MIR141	NAA60	LOC100505622	SNHG1	LOC728175	INPPL1
PRR13	RMI2	LBH	RASGRP2	ACSL1	VSIG2
RDH5	MAZ	C2orf73	TM7SF2	LOC731424	DCPS
CEBPE	ZNF48	SCLY	LTBP3	LINC01093	SLC2A3
IRF9	PRR14	BCL2L1	CD248	HLA-A	FOXJ2
KLF13	PLLP	TGIF2-C20orf24	PITPNM1	ACTB	CLEC2D
PSMA4	CKLF-CMTM1	SNORA71C	CD3D	CCL24	ETV6
DDX11L10	MTSS1L	PLCG1	CXCR5	NEK6	HELB
MIR5587	HCCAT5	TP53TG5	MIR3656	FCN1	TPCN1
STUB1	ZNRF1	PMEPA1	ENO2	ENO1	SPPL3
CCDC78	ZCCHC14	NAT6	HDAC7	MIR4254	KDM2B

NME3	MC1R	ZNF639	MFSD5	FHL3	ABCB9
SNORA64	OVCA2	SPINK2	OAS1	MIR4257	SNRNP35
SNN	RASD1	LRRC14B	RHOF	IL6R	SLC15A4
YPEL3	MIEF2	MZB1	B3GNT4	GBAP1	EP400
CETP	SNORD4A	N4BP3	ARL6IP4	ETV3L	N4BP2L1
CYBA	MIR152	CD83	SNORD102	FLJ23867	LINC00598
SNAI3	MYCBPAP	HCP5	CHAMP1	NLRP3	LCP1
RNF166	CYB561	LTB	RPS29	PFKFB3	TRIM13
CTU2	GPR142	LOC442497	GPR68	ZNF22	DHRS4L2
KCTD11	ZACN	CARD11	EVL	HK1	TMED10
SNORD10	SIGLEC15	STK17A	CRIP2	STK32C	FOXN3
CD68	TSHZ1	MIR4649	BAHD1	TALDO1	PAPOLA
MPDU1	CACTIN-AS1	C7orf72	C15orf62	IFITM10	SLC25A29
SNORD4B	RAX2	STAG3L1	ELL3	MRPL23-AS1	PLD4
MIR144	NRTN	GPC2	NMB	OSBPL5	VPS18
MIR4726	SYCE2	ARHGEF35	MRPL28	LOC221122	MIR4310
SPATA32	PALM3	MIR4672	TMEM8A	BEST1	SNAPC5
LRRC37A4P	B9D2	FAM163B	FAM173A	SAC3D1	CLN6
MIR3614	KPTN	PIM2	TMEM204	AP5B1	SEMA4B
SNHG16	KDELR1	FOXP3	IL32	ANKRD13D	SNRNP25
METTL23	RNF144A-AS1	CXorf65	HMOX2	MIR326	ATP6V0C
TMC6	EMILIN1	ZNF691	LITAF	AMICA1	PDPK1
TMC8	TRABD2A	FCRL3	MYLPF	PLEKHG6	LOC652276
ARHGDIA	PDK1	MIR3916	ZNF747	CELA1	SLX4
ROCK1P1	ABCB6	C10orf12	CTF1	ACVR1B	C16orf52
PTBP1	NOP56	MIR1260B	ESRP2	TESC	NPIPB5
MIR4745	SNORD110	HTR3A	ZFPM1	LAMP1	APOBR
LPPR3	E2F1	APOBEC1	RNF167	TFDP1	STX1B
R3HDM4	TOX2	ATG101	TNFSF12	LTB4R2	KAT8
CNN2	TP53RK	TESPA1	SOX15	LTB4R	AMFR
HMHA1	MIR3196	GPR33	CCDC42	ADCY4	TK2
POLR2E	LINC00176	COX8C	SREBF1	CALML4	ATP6V0D1
CIRBP	PCMTD2	MIR369	CDK5R1	WFIKKN1	HAS3
MOB3A	PDE9A	KIAA0125	MLLT6	PTX4	ST3GAL2
EEF2	COL18A1-AS2	SERINC4	STARD3	CEMP1	CMIP
ZBTB7A	PRMT2	PFN1	PGAP3	CLDN9	ABR
LOC100128573	MIR1306	APCDD1	THRA	ROGDI	SLC43A2
ANGPTL6	LIMK2	F2RL3	NR1D1	NUPR1	SERPINF2
STX10	FAM118A	PPP1R14A	TNS4	SULT1A1	CLUH
MIR24-2	NT5DC2	MIR4531	HSD17B1	ZNF688	TMEM11
MIR23A	AP2M1	IKZF2	FAM215A	PRSS8	MIR4522
CD97	SNORD66	LINC00494	PPP1R9B	NOD2	GHDC
MIR3188	KLF3	LINC00163	MSX2P1	GPR97	HDAC5
MIR5196	PTGER4	IGLL5	OR4D2	SLC22A31	ARHGAP27
TYROBP	IL6ST	A4GALT	SMARCD2	FAM101B	PLEKHM1
GMFG	SERINC5	TREX1	ICAM2	SPNS3	ARL17B
TGFB1	EGR1	LECT2	FOXJ1	CHD3	SNX11
CD79A	GRPEL2	PRL	OXLD1	TRIM25	GNGT2
VASP	GNB2L1	MIR4462	STRA13	MIF4GD	COL1A1
EMP3	RXRB	LINC01013	CD7	SNORD1A	LINC00483

LENG1	PRKAR1B	BLK	NDUFV2	TIMP2	FTSJ3
MIR5696	TBRG4	NKX6-3	NFATC1	BAIAP2-AS1	PLEKHM1P
LOC100499194	SNORA5B	LYN	GZMM	AATK	TTYH2
GPR17	C7orf61	MIR4667	KLF16	TMEM105	MRPS7
ZFAND2B	ZNF862	TAL2	ABHD17A	P4HB	TEN1-CDK3
MIR663A	ASIC3	PTGS1	ZFR2	MAFG	MIR4740
RBM38	MIR1205	MIR200A	ATG4D	MAFG-AS1	PDE6G
CTSZ	SLURP1	SLC25A34	SAMD1	LRRC45	TBCD
TNFRSF6B	GPIHBP1	PQLC2	RASAL3	LDLRAD4-AS1	ATP5A1
MIR1914	ZBTB34	CNR2	SSBP4	ZNF516	IZUMO4
MIR647	TTC16	GSTM4	LPAR2	FSTL3	SPPL2B
UCKL1-AS1	C9orf106	KCNA3	U2AF1L4	PRSS57	TMPRSS9
ITGB2	PPP1R3F	FCRL6	CAPN12	AZU1	GNG7
ADORA2A	SLC10A3	CTSE	EGLN2	ELANE	GNA15
RAC2	TTLL10	ACBD3	CALM3	TIMM13	NFIC
CYTH4	B3GALT6	GDI2	FLT3LG	NCLN	UBXN6
RNU86	UBE2J2	VENTX	NOSIP	UHRF1	TICAM1
TRABD	MMP23A	SBF2-AS1	LOC100133985	PCP2	KDM4B
PPM1M	SLC35E2	CTNND1	SH2D6	RAB3D	MLLT1
ZNF595	THAP3	ZFP91	DUSP2	PRKACA	VAV1
MYL5	CLSTN1	CYB561A3	ANKRD23	ASF1B	MCOLN1
SH3BP2	IFFO2	LRP5	ZAP70	ARMC6	PNPLA6
RAB33B	CAPZB	FLJ42102	NCK2	FXYD1	CD320
RGS14	LYPLA2	PCF11	PDCD1	PAF1	ZGLP1
GRK6	PAFAH2	GNB3	SOX12	B3GNT8	RAVER1
CBY3	YTHDF2	WIBG	FASTKD5	ZNF526	MAN2B1
SNORD96A	HPDL	RNF41	NOL4L	LILRB3	ANKLE1
LOC285819	JAK1	PRIM1	C20orf144	KHK	SLC27A1
TNF	FUBP1	HVCN1	FAM83D	FOSL2	ELL
RNF5	CYB561D1	FAM109A	STMN3	FLJ42351	HAMP
WDR46	CD2	BCL7A	SLC2A4RG	PNKD	ACTN4
MIR4648	PSMB4	DLEU1	SAMD10	SLC11A1	BLVRB
MIR589	SNAPIN	DLEU7-AS1	KRTAP12-4	HDAC4	C19orf54
ARPC1B	SLC39A1	APEX1	SSR4P1	MGC16025	EXOSC5
MIR93	FAM189B	TSSK4	RTN4R	MIR2467	PINLYP
MIR4658	ARHGAP30	YLPM1	C22orf15	CD93	CLASRP
GIGYF1	LINC00626	TRAF3	TBC1D10A	CST7	NAPSA
RNU6-33P	RASSF5	EXOC3L4	MFNG	SNORD12B	LAIR2
DOK2	ARID4B	NIPA2	MICALL1	LSM14B	PPP1R12C
PLEC	CASC10	SEMA7A	CSNK1E	NTSR1	CCDC106
MIR661	DNAJB12	CIITA	SUN2	TPD52L2	YWHAQ
SIT1	FAM213A	CD19	PIM3	DNAJC5	TTC7A
GLIPR2	AGAP11	SLC12A3	SBF1	PTTG1IP	PCGF1
FAM27B	IFIT5	CX3CL1	IL17RC	CECR6	KCMF1
MIR4673	MGEA5	CCL17	RNF123	YWHAH	CAPG
FUT7	KCNIP2-AS1	PLA2G15	CISH	GALR3	NMI
DPH7	FUOM	BANP	ABHD14B	TSPO	SP3
.	NLRP6	LINC00304	TLR9	CAMK1	LRRFIP1
LINC01342	RASSF7	GP1BA	RAB43	PFKFB4	PCED1A
ACAP3	KCNJ11	ABHD15	ADIPOQ-AS1	ALAS1	RRBP1

GNB1	CD5	CCL16	RTP4	TKT	ZNF341
C1orf174	TTC9C	MSI2	LINC00887	CD200R1	MIR499B
MST1P2	GPR137	UBE2O	MIR4457	TADA2B	PREX1
CDC42	SNX15	DIRAS1	TCF7	FGFBP2	RNF114
MDS2	ZNHIT2	ELOF1	ECSCR	PELO	NPEPL1
LDLRAP1	FRMD8	FAM129C	RNF44	LMNB1	MX1
UBXN11	FAM89B	CD22	TSPAN17	HRH2	RRP1
LAPTM5	PCNXL3	MIR4530	PRR7-AS1	NOP16	BID
PTP4A2	CTSW	APOC4-APOC2	NHP2	PRELID1	DGCR2
ZC3H12A	BANF1	SPIB	HIST1H3A	MAML1	UBE2L3
BTBD19	CLCF1	LILRA4	LY6G6E	MIR1229	MAPK1
PLEKHO1	PPP6R3	LOC90784	AGER	VWA7	ADORA2A-AS1
CDC42SE1	TMEM123	STARD7	CUTA	TUBE1	ASPHD2
CHTOP	ATN1	B3GNT7	RPS10	DAGLB	TOM1
PBXIP1	C12orf57	ESPNL	DEF6	LRRC4	MCM5
ZBTB7B	GALNT6	KLHL30	PI16	LOC100506585	LOC100506472
SLAMF8	SP1	CSNK2A1	CCND3	SLC45A4	C22orf46
FAIM3	ITGA5	LOC149950	DNPH1	TOP1MT	POLDIP3
JMJD4	ESYT1	SOGA1	BCLAF1	MIR937	RIBC2
FUT11	MYL6B	NCOA3	HECA	WASH1	TTC38
C10orf91	NAB2	LINC00162	RPS2P32	LOC642236	GRAMD4
ATHL1	CTDSP2	P2RX6P	MYO1G	LCN2	PANX2
SIGIRR	RASSF3	SMARCB1	REPIN1	LINC00963	PLXNB2
MIR210HG	C12orf74	LRRC75B	NOS3	NACC2	EGOT
IRF7	ELK3	AP1B1	PDLIM2	NPDC1	VHL
SNORA52	P2RX4	RNF215	BIN3-IT1	TUBB4B	NUP210
TOLLIP-AS1	PITPNM2	RNF7	PTPLAD2	NADK	ZNF589
CD81	C12orf65	HPS3	SUSD3	FCN3	PLXND1
EML3	GOLGA3	ST6GAL1	PTRH1	DENND2D	FXR1
HRASLS2	ELF1	MAP3K1	ST6GALNAC6	C1orf162	TRA2B
PYGM	DHRS12	C5orf27	DPM2	GNRHR2	XXYLT1
MAP4K2	CLN5	SLC23A1	PTGES2	S100A11	LINC00885
SCYL1	UBAC2	CXXC5	RALGDS	PSAP	ZDHHC19
SIPA1	PCK2	CD74	C9orf163	LOC440028	NRROS
C11orf68	MGAT2	ERGIC1	C9orf139	SAA2	GAK
CARNS1	C14orf182	ATP6V1G2	NAA10	TP53I11	FBXL5
PTPRCAP	SRSF5	FGD2	SNORA56	SPI1	TMEM165
AIP	ABCD4	RPS6KA2-IT1	NOC2L	MPEG1	IRF2
CDK2AP2	LINC01220	GPR31	SDF4	BATF2	LOC728613
MYEOV	IRF2BPL	WIPI2	TAS1R3	ALDH3B1	PAIP2
SNORD15A	YY1	LIMK1	DVL1	RELT	SOX30
BCL9L	PPP2R5C	STAG3	MXRA8	UCP2	LMAN2
TAPBPL	NUDT14	SH2B2	TMEM240	TMEM126A	RREB1
LPAR5	BRF1	ADAMDEC1	CDK11B	CTSC	JARID2
PTGES3	LOC283683	RNF122	SLC35E2B	CDK2	PPP1R18
TMPO	SRP14	WHSC1L1	CDK11A	KIAA0226L	MDC1
ORAI1	TMOD2	PLEKHF2	TNFRSF14	KLHL33	C6orf25
MORN3	ARPP19	ZFAT-AS1	EFHD2	LINC00641	SNORD52
UBC	USP3	ADCK5	ZBTB17	C15orf52	BAK1
STK24	SMAD3	LOC286297	NBPF1	MIR4312	MAD2L1BP

RASA3	ANP32A-IT1	BICD2	GALE	ST20-MTHFS	RRAGD
ACOT2	SCAMP2	C9orf9	RPS6KA1	CHD2	GPER1
CCDC88C	CIB2	VAV2	LOC644961	CLCN7	FTSJ2
DEGS2	AEN	LCN10	SNHG3	FLJ42627	NUDT1
INF2	IDH2	LCN6	RAB42	IGSF6	IQCE
GPR132	CRTC3	LOC100128593	CAP1	LOC554206	AMZ1
FBXL22	DNM1P46	LCN8	CCDC17	IRF8	FBXL18
CSK	NME4	LCN15	FAM159A	CBFA2T3	EIF2AK1
MORF4L1	RAB11FIP3	ENTPD8	RSBN1	DEF8	ZDHHC4
ISG20	TSR3	MIR429	NBPF10	SMCR8	RSBN1L
SNORA10	GNPTG	PLCH2	ANXA9	ARGFXP2	PILRA
PRSS30P	HN1L	ZBTB48	BGLAP	LINC00910	PPP1R35
HCFC1R1	SYNGR3	FLJ37453	PVRL4	LOC644172	IRF5
UBALD1	CCDC64B	CROCCP2	POGK	PECAM1	ZNF212
SMG1P1	NLRC3	LOC100506801	KIF21B	RHBDF2	DNAJB6
NSMCE1	DNASE1	TRNP1	PARP1	TK1	SLC25A37
LAT	CREBBP	WASF2	GUK1	LINC00482	RNF139
LOC606724	ZC3H7A	KHDRBS1	IBA57	BAHCC1	UBE2R2
BOLA2B	SLC7A5P1	HDAC1	CELF2	PPP1R27	MSMP
C16orf54	ZNF768	SMAP2	PIP4K2A	FCER2	OSTF1
SLC7A6	FUS	SLC2A1	HNRNPF	PRAM1	C9orf89
MIR5189	PYCARD	SIKE1	C10orf55	OR7C2	SUSD1
MVD	MIR548AE2	MUC1	ZMIZ1	RAB8A	GAPVD1
PIEZO1	CCDC102A	MPZ	TRIM8	HSH2D	PPP2R4
APRT	CMTM3	KIAA0040	PHRF1	KLF2	QRFP
ANKRD11	PARD6A	LINC01136	SLC25A22	IFI30	POMT1
LOC100287036	KLHL36	LOC148696	PIDD1	CD3EAP	BRD3
WDR81	FAM92B	DEGS1	AP2A2	LOC100129083	UAP1L1
P2RX5-TAX1BP3	SNAI3-AS1	DUSP5P1	KCNQ1	SPOPL	SLC34A3
ATP2A3	CDH15	RAB4A	ILK	GPBAR1	SASH3
ARRB2	RPL13	OPN3	DCHS1	TRERNA1	P2RY8
ACAP1	CHMP1A	DDIT4	MARK2	IFNGR2	LOC100130417
LOC284023	ZNF276	IFIT3	COX8A	LOC284837	C1orf200
GRAP	TCF25	LHPP	TRPT1	FBXW4P1	HENMT1
UNC119	PAFAH1B1	PRAP1	VEGFB	BIK	MAB21L3
SNORA21	PLD2	PAOX	CCDC88B	KIAA0930	ATP1A1
MIR4316	SLC16A13	TMEM80	CAPN1	UPK3A	PHLDA3
FLJ45079	SLC35G6	CD82	KCNK7	ZBTB47	TMCC2
SYNGR2	TNFSF13	GYLTL1B	MIR4489	CCR1	FCAMR
CYTH1	PLD6	MS4A5	FIBP	PRKCD	OR2W5
LGALS3BP	RAI1	TMEM132A	TSGA10IP	LPCAT1	C10orf10
C17orf89	SMCR5	MTA2	YIF1A	SNX18	ARHGAP22
SMAD7	FAM83G	RBM14-RBM4	RBM14	MXD3	SNCG
FGF22	PROCA1	POLD4	RBM4	DOK3	NSMCE4A
RNF126	TRAF4	ACY3	NDUFS8	DUSP22	KCNQ1DN
TMEM259	TP53I13	SIDT2	ARRB1	C6orf136	RCN1
ABCA7	LINC00672	DDX6	FXYD6-FXYD2	DDAH2	MS4A12
DAZAP1	LEPREL4	METTL20	H2AFX	MIR4655	DAGLA
SCAMP4	FAM171A2	TUBA1A	UBASH3B	RNF216-IT1	KRTAP5-9
CSNK1G2	MAP3K14	BIN2	FLI1	SNX10	C11orf53

BTBD2	LRRC46	ITGB7	TPI1	NCF1B	ST3GAL4
MKNK2	DYNLL2	SHMT2	LOC283335	LAT2	LOC574538
JSRP1	PRR29	PPTC7	SPRYD3	NCF1	LOC100335030
AES	C17orf58	TCTN1	ZBTB39	NCF1C	AQP6
MFSD12	AMZ2	ERP29	STAT6	PILRB	SDR9C7
SNORD37	RPL38	LINC00173	ARHGAP9	SYPL1	MIR487B
RPL36	NAT9	VPS37B	MBD6	PIK3CG	MIR4309
ALKBH7	ARMC7	LOC100507091	RAP1B	MTPN	FES
PPAN-P2RY11	UBALD2	FOXO1	ANKRD13A	RAB11FIP1	C16orf59
SNORD105	QRICH2	HNRNPC	SH2B3	SLC2A6	ZG16B
MIR1238	PRCD	PPP1R3E	RPL6	TOR4A	PDZD9
ILF3-AS1	LOC100996291	KHNYN	SLC8B1	MIR3202-1	CORO1A
ACP5	SOCS3	C14orf183	RAB35		ASGR2
GADD45GIP1	DUS1L	NIN	LINC01089		LRRC75A-AS1
RFX1	RPS15	KIAA0247	SETD1B		SNORD65
DDX39A	LSM7	RIN3	MLXIP		JUP
NR2F6	GNA11	XRCC3	NCOR2		VPS25
ARRDC2	SMIM24	LOC283710	ULK1		MPO
LSM4	TBXA2R	BMF	FBRSL1		AATK-AS1
GMIP	CACTIN	GTF2A2	MIR548AN		ZNF750
MIR641	MATK	SNORD18C	PNP		RPL17
RAB4B-EGLN2	STAP2	TM2D3	REC8		PALM
RPS19	TNFAIP8L1	MAPK8IP3	ELMSAN1		TMEM150B
ARHGEF1	FEM1A	CDIP1	FOXN3-AS1		SLC1A4
CIC	RANBP3	USP7	SIVA1		AMMECR1L
RPL18	CLPP	C16orf72	CDCA4		CPXM1
SNORD33	ARHGEF18	GGA2	MTA1		UBOX5-AS1
NDUFA3	MAP2K7	IL21R	C15orf39		C20orf203
KLF11	RAB11B	RABEP2	SNX33		MIR3646
ZNF513	C19orf66	TRIM72	PDIA2		ZNF295-AS1
MIR5192	LPHN1	FAM65A	AXIN1		UMODL1
C2orf68	DNAJB1	DPEP2	LOC100134368		TFF1
SEMA4C	ANO8	USP10	CAPN15		MLC1
PSD4	NDUFA13	IL17C	RAB40C		SHANK3
HNRNPA3	ATP13A1	ACSF3	RHOT2		GPR27
CTDSP1	ZNF101	YWHAE	RHBDL1		DPPA4
MIR26B	IGFLR1	MYO1C	NARFL		CHST13
CDC25B	LGALS7	SCARF1	RPUSD1		MGLL
ZNF217	MED29	SERPINF1	BAIAP3		CYTL1
ZBP1	AKT2	P2RX5	EME2		PDLIM4
PTK6	HNRNPUL1	GRAPL	RAB26		FLT4
C20orf195	CCDC97	MAP2K3	TRAF7		SERPINB6
RTEL1-TNFRSF6B	ERF	RPL23A	DNASE1L2		LTA
UCKL1	PRKD2	NEK8	ERVK13-1		LY6G6D
RGS19	NAPA-AS1	ZNF207	TCEB2		TREML4
C21orf33	CCDC155	DHX58	TNFRSF12A		GNA12
AIRE	PIH1D1	KANSL1	ZSCAN10		TNRC18
CECR5	ALDH16A1	ABI3	LINC00921		SNORD93
LINC00896	PRRG2	LUC7L3	CORO7		TRIP6
MIR548J	MIR5088	CD79B	VASN		ZC3HAV1L

MYH9	CNOT3	GNA13	NMRAL1		GIMAP5
CMTM7	LENG8	SUMO2	MIR3180-4		GPR124
CTNNB1	HSPBP1	GRB2	IL4R		RAB2A
CCR2	NAT14	CYGB	XPO6		LINC00051
GPX1	A1BG	C17orf99	TBX6		PSCA
TUSC2	UBE2M	HEXDC	ZNF771		RGAG4
WDR82	SNORA80B	C17orf62	DCTPP1		SCNN1D
MIRLET7G	PREB	TCF3	ITGAL		CPSF3L
GLYCTK	CCT4	DOT1L	ZNF629		LOC148413
MUC4	FAM136A	FZR1	ADCY7		RER1
MIR922	MRPL53	C19orf10	SNX20		MEGF6
MFSD10	BIN1	SLC44A2	CPNE2		KCNAB2
NOP14	LIMS2	ILF3	SLC7A5		H6PD
FLJ36777	SF3B1	ZNF791	ZNF469		SLC25A33
DANCR	FAM117B	MIR639	SPATA2L		SRM
TAF9	EIF4E2	TPM4	VPS9D1-AS1		ESPNP
TNFAIP8	DGKD	EPS15L1	MNT		CROCC
CDC42SE2	RTP5	MED26	ENO3		RCC2
TMCO6	TGIF2	FCHO1	SPAG7		ECE1
MGAT1	PKIG	PLEKHF1	ZBTB4		SLC9A1
LST1	TMEM189-UBE2V	MAP4K1	KDM6B		NCDN
C6orf47	DIDO1	RINL	TMEM107		MAP7D1
HSPA1A	SLC17A9	ZFP36	PIK3R5		SH3D21
ZBTB12	LIME1	MIA	MAGOH2		STK40
TAP2	ABHD16B	MIA-RAB4B	MED9		SNORD45A
TAPBP	IL10RB-AS1	POU2F2	USP22		AMPD2
SLC35B2	CBR3	ZNF296	KCNJ18		CD58
MICAL1	TRAPPC10	PPP1R37	FLJ36000		CERS2
ZBTB24	ICOSLG	STRN4	ERAL1		FAM63A
TSPYL1	TXNRD2	CD37	FLOT2		DENND4B
TAGAP	MED15	TEAD2	GIT1		JTB
WTAP	CRKL	FUZ	STAT5A		TPM3
MAFK	THAP7	MIR4749	ATXN7L3		PMVK
LFNG	PATZ1	MIR4750	FAM117A		SHC1
HNRNPA2B1	KCTD17	IL4I1	RSAD1		HDGF
PGAM2	SSTR3	NUP62	SLC16A5		F11R
TRIM73	CDC42EP1	LINC00309	HN1		MAPKAPK2
C7orf43	LGALS1	LGALSL	UNC13D		WDR37
SAP25	TOMM22	MOB1A	WBP2		FBXO18
TMUB1	APOBEC3F	C2orf81	TRIM65		FRAT2
ZNF395	TNRC6B	CNNM4	PGS1		ZFYVE27
LY6E	EP300	MAP4K4	RPTOR		CUEDC2
PUF60	NHP2L1	MYO7B	MRPL12		TAF5
CD274	OGFRP1	CCNT2	PCYT2		PRDX3
SEMA4D	CDPF1	CYTIP	ASPSCR1		EPS8L2
FGD3	CRELD2	ITM2C	CCDC57		POLR2L
AKNA	IL17REL	INPP5D	SECTM1		CCDC86
FAM102A	DENND6B	EIF6	FOXK2		HNRNPUL2-BSCL2
NTMT1	MIOX	PTPN1	BCL2		C11orf84
FAM78A	GORASP1	ADNP	TPGS1		STIP1

SDCCAG3	CSRNP1	SRMS	BSG		MIR1237
NOTCH1	CCRL2	C21orf128	CFD		SPDYC
C9orf142	SHISA5	SIK1	WDR18		TIGD3
NSMF	WDR6	C21orf67	GRIN3B		KDM2A
DDX11L1	KLHDC8B	ADARB1	C19orf24		STARD10
PUSL1	C3orf62	DERL3	GAMT		ARHGEF17
SSU72	RRP9	LIF	C19orf25		FXYD2
TNFRSF1B	TNNC1	GGA1	REXO1		C2CD2L
SEPN1	FOXP1	PDXP	AP3D1		ST3GAL4-AS1
FGR	ZBTB11	MGAT3	SF3A2		CCND2
PLK3	CDV3	SGSM3	OAZ1		HEBP1
RPE65	TBL1XR1	TNFRSF13C	MPND		LETMD1
RBM15	GP5	CENPM	PSPN		ZNF385A
CHI3L2	CTBP1-AS2	CYB5R3	KHSRP		SMARCC2
TNFAIP8L2-SCNM	LETM1	IQSEC1	GPR108		CNPY2
LYSMD1	MXD4	SH3BP5	PEX11G		EID3
RXFP4	RNF4	TGFBR2	ICAM3		FBXO21
ARHGEF2	CNOT6L	CX3CR1	KEAP1		RNF10
C1orf105	PDCD6	LIMD1	CDKN2D		SETD8
PTPN7	MIR4635	IP6K2	DHPS		STX2
LINC01132	CLPTM1L	RHOA	LOC284454		PGAM5
CUL2	GPBP1	RPL24	DCAF15		TEX30
SRGN	MBLAC2	CNBP	GIPC1		GAS6-AS1
ANXA11	IRF1	MSL2	PGLYRP2		GAS6
TSPAN14	JADE2	MBNL1	OCEL1		TMEM55B
C10orf95	SLC35A4	SKIL	ABHD8		LINC00648
CALHM3	HDAC3	SH3TC1	MAP1S		ZNF410
PWWP2B	FCHSD1	RHOH	MAST3		PGF
TUBGCP2	CCDC69	HNRNPD	KIAA1683		BEGAIN
ZNF511	UBLCP1	PLAC8	FKBP8		ADSSL1
ECHS1	FAM65B	KIAA0922	GATAD2A		C15orf56
MIR3944	HLA-F	PP7080	CAPNS1		CHAC1
HRAS	C6orf48	SUB1	SNRPA		DNAJA4
CEND1	EHMT2	GZMK	ETHE1		ST20
PNPLA2	EGFL8	MATR3	BCL3		ZNF592
CD151	PSMB8	UBE2D2	TRAPPC6A		NHLRC4
TOLLIP	PSMB9	LOC100268168	PPP5C		PIGQ
TSPAN32	DAXX	SLC35B3	CCDC9		C16orf91
SLC22A18AS	MTCH1	NHLRC1	SPHK2		NUBP2
RHOG	TBCC	HIST1H1A	DBP		RPL3L
C11orf94	ZNF318	HIST1H2AE	PPP1R15A		TBL3
DGKZ	BACH2	HIST1H2BK	BAX		ZNF598
MIR4688	FYN	NFKBIL1	SNRNP70		PKD1
TMEM179B	C7orf50	HLA-DMA	IRF3		E4F1
SLC25A45	GPR146	ZBTB22	PRMT1		KCTD5
EHBP1L1	PSMG3	TREML3P	ADM5		PRSS27
SNX32	ZNF853	TFEB	CLDND2		PAQR4
EFEMP2	RASA4CP	STX7	MYADM		CLDN6
LRFN4	AEBP1	ZC3H12D	TFPT		TFAP4
RAD9A	EIF4H	OSTCP1	TMC4		CORO7-PAM16

GPR152	TECPR1	DBNL	PPP6R1		PAM16
ARAP1	CNPY4	POLM	U2AF2		ZNF500
LOC100652768	TSC22D4	IKZF1	MZF1		SULT1A2
IL10RA	GNB2	GTF2I	DNMT3A		DOC2A
LINC00167	TRIM56	TMEM120A	SNX17		ALDOA
LOC100288778	MIR4467	PVRIG	SOCS5		GDPD3
NINJ2	ARHGEF5	LOC100630923	DGUOK		TBC1D10B
TNFRSF1A	GIMAP1	ORAI2	POLR1A		FBRS
IFFO1	ATG9B	RASA4	INPP4A		SETD1A
NOP2	SGK223	ZC3HAV1	GYPC		HSD3B7
PCED1B-AS1	MFHAS1	LOC728743	OSGEPL1		ZNF646
MIR4701	FAM167A	WDR86-AS1	RNPEPL1		DNAJA2
TUBA1C	BIN3	CLN8	SRXN1		CBFB
SPRYD4	TNFRSF10A	SORBS3	FKBP1A		NRN1L
DDIT3	SMIM19	PPP2R2A	C20orf27		PRMT7
AGAP2-AS1	ST3GAL1	PLEKHA2	CENPB		VAC14
VPS29	TMEM249	YWHAZ	RASSF2		GINS2
PXN	CD72	UHRF2	PLAGL2		COX4I1
AKT1	TLN1	B4GALT1	RBM39		LOC400558
RPS27L	PHF2	LOC100133920	C20orf24		SNORD68
PIF1	ENDOG	ATP6V1G1	SLC35C2		DPEP1
NEIL1	LRRC8A	ST6GALNAC4	NELFCD		CDK10
PSTPIP1	FAM73B	NAIF1	OSBPL2		ITGAE
ZNF710	ASB6	FNBP1	SLCO4A1		ALOX12
FURIN	PRRC2B	RAPGEF1	COL9A3		DVL2
DDX11L9	CEL	CACFD1	TCFL5		ELP5
FAM195A	PMPCA	QSOX2	BIRC7		PLSCR3
JMJD8	EDF1	C9orf173	GMEB2		TMEM102
CHTF18	PNPLA7	NOXA1	ARFRP1		POLR2A
UBE2I	EBP	EHMT1	TCEA2		TNFSF12-TNFSF13
SPSB3	MSN	ABCD1	SON		PER1
MIR4516	IRAK1		RUNX1		MIR33B
MLST8	GDI1		LOC100133286		NUFIP2
BRICD5	MRPL20		CRYAA		SUZ12P1
AMDHD2	VWA1		PFKL		ASB16
SRRM2	GABRD		LRRC3		DCAKD
MGRN1	GPR157		SUMO3		EPN3
CARHSP1	TMEM201		DIP2A		MAP3K3
PRM3	CD164L2		SLC25A1		PSMD12
SULT1A3	AHDC1		TRMT2A		UNK
MAPK3	SRSF4		LOC388849		USP36
PRSS36	C1orf216		HIC2		CANT1
MT1X	AKIRIN1		RAB36		RNF213
E2F4	PPM1J		BCR		BAIAP2
ELMO3	GJA8		ZNF70		CCDC137
HSBP1	MEX3A		CHCHD10		ARL16
COTL1	C1orf27		TPST2		NPB
GSE1	RAB29		OSM		RAC3
CDT1	LEFTY1		APOL6		GPS1
TRAPPC2L	TOMM20		DDX17		B3GNTL1

SPIRE2	ASB13		SHISA8		EMILIN2
P2RX1	C10orf99		CYP2D6		LDLRAD4
CAMTA2	PTEN		CYP2D7P		RNF138
ACADVL	EXOSC1		LDOC1L		LOC100131655
TMEM88	MOB2		LOC90834		GPX4
MFAP4	TRIM22		TUBGCP6		C19orf26
CRLF3	DENND5A		HDAC10		MUM1
LASP1	CAPRIN1		KLHDC7B		PCSK4
RARA	KBTBD4		TRNT1		MBD3
FMNL1	INTS5		ACAA1		PLEKHJ1
SLC16A6	PRDX5		VIPR1		LMNB2
MGC16275	CNIH2		CCDC12		SGTA
RAB37	TMEM134		NBEAL2		PIP5K1C
SLC9A3R1	SUV420H1		MIR1226		DAPK3
CBX4	USP35		MST1		PIAS4
SLC38A10	TIRAP		RASSF1		SAFB2
SLC16A3	AEBP2		C3orf18		SAFB
CSNK1D	ATP5B		PARP3		LONP1
METRNL	C12orf45		FAM208A		TIMM44
MBP	RITA1		PODXL2		ZNF414
MIR3187	MAP1LC3B2		EIF4G1		EIF3G
MED16	EBPL		DGKQ		S1PR2
ARID3A	CBLN3		MAEA		MIR199A1
SBNO2	MAP4K5		MIR943		TMED1
STK11	FLJ31306		MIR4800		LDLR
ATP5D	JAG2		ADD1		NACC1
MIDN	HERC2P7		TBC1D14		ZSWIM4
UQCR11	SRP14-AS1		SLC12A7		MIR181C
CSNK1G2-AS1	SPTBN5		FYB		PKN1
MIR1227	PKM		SLC22A5		PTGER1
C19orf35	STOML1		TMEM173		TECR
LINGO3	IMP3		WDR55		BRD4
C19orf71	NTHL1		DIAPH1		CHERP
HMG20B	SMG1P3		LCP2		USHBP1
MAP2K2	TUFM		PHYKPL		BST2
SH3GL1	KIF22		RUFY1		C19orf60
TNFSF14	PAGR1		MGAT4B		COPE
STXBP2	SMG1P5		SQSTM1		NFKBID
MCEMP1	N4BP1		FAM50B		PAK4
HNRNPM	GPR56		HCG18		SMG9
MYO1F	RLTPR		PPP1R10		NKPD1
P2RY11	ACD		DDX39B		DMWD
DNM2	NFATC3		SNORD84		MYPOP
TMEM205	VPS4A		PRRC2A		FKRP
RAD23A	MAP1LC3B		APOM		ZC3H4
MYO9B	KLHDC4		SAPCD1		C5AR2
JAK3	PITPNA		BYSL		RUVBL2
PIK3R2	SLC25A11		TNFAIP3		LIN7B
ISYNA1	DLG4		GET4		FCGRT
UPF1	TOM1L2		COX19		RCN3

NR2C2AP	SNORD42B		TMEM184A		PRR12
GRAMD1A	SYNRG		MAD1L1		RRAS
USF2	LOC440434		FOXK1		MIR4751
ZNF574	FAM134C		HOXA4		MIR99B
PTGIR	HILS1		LOC541473		ZBTB45
PTOV1	GALK1		PDAP1		CHMP2A
NR1H2	TMEM235		COPS6		FAM179A
SIGLEC9	SLMO1		PCOLCE		MSH6
LAIR1	TXNL4A		SRRT		B3GNT2
ZNF524	FUT6		POLR2J		LOC339807
ZNF787	EVI5L		POLR2J3		DOK1
HPCAL1	SNAPC2		POLR2J2		TGOLN2
DDX11L2	FDX1L		CASP2		ZC3H8
PTPN18	KRI1		EPHA1		RALB
CLK1	SMARCA4		GIMAP8		NIFK
CASP10	KANK2		GIMAP4		POLR2D
CATIP	TNPO2		GIMAP1-GIMAP5		RBMS1
DUSP28	CALR		SLC4A2		STK17B
GPR35	TRMT1		FASTK		PPIL3
D2HGDH	MRI1		REEP4		CAB39
PANK2	MIR181D		AGO2		CHRNG
GZF1	IL27RA		THEM6		FAM110A
RBM12	SYDE1		ZFP41		HSPA12B
CTSA	AP1M1		GLI4		MAPRE1
LOC100131496	KLHL26		RHPN1		VAPB
MIR5095	CRTC1		ZC3H3		SLMO2
LINC00659	LSM14A		GSDMD		TAF4
OGFR	FXYD3		EEF1D		CABLES2
HELZ2	FAM187B		TIGD5		YTHDF1
ZGPAT	MAG		BREA2		RTEL1
MORC3	HNRNPL		NRBP2		OPRL1
PDXK	CLEC11A		GRINA		ABCG1
C21orf2	MBOAT7		DGAT1		SLC37A1
ITGB2-AS1	RPS9		VPS28		WDR4
FAM207A	ISOC2		GPT		AGPAT3
SLC19A1	LOC375196		RECQL4		PWP2
S100B	MCFD2		FAM27C		LOC642852
IL17RA	CALM2		SH2D3C		COL18A1
SEPT5-GP1BB	GMCL1		PKN3		MCM3AP
MIR4761	SNRPG		USP20		YBEY
MIR185	LOC654342		C9orf69		COMT
DDTL	NEURL3		GPSM1		TANGO2
PISD	TMEM177		DNLZ		DGCR8
RPS19BP1	HS6ST1		CARD9		PI4KAP2
SMDT1	MIR4784		TRAF2		YDJC
CERK	CFLAR		C8G		CCDC116
BRD1	PKI55		PTGDS		PPM1F
SELO	LOC643387		CLIC3		MORC2
SLC6A6	NEU4		ABCA2		EIF3D
GNAI2	TBC1D20		NDOR1		SH3BP1

HYAL2	DDRGK1		RNF113A		H1F0
ZMYND10	SNX5		XPNPEP2		ATF4
ABHD14A	ENTPD6		SRPK3		CHADL
RPL29	ADA		FAM3A		C22orf34
LINC00696	SPATA25		TMEM88B		ZBED4
TRIM59	C20orf166-AS1		UBIAD1		CPT1B
TNK2	ZBTB46		MAN1C1		ARSA
MFSD7	C2CD2		LINC01225		BHLHE40-AS1
LOC100130872	LINC00319		CCDC23		TADA3
CTBP1	CSTB		HYI		RPUSD3
TMEM129	SCARF2		LINC01144		PRRT3-AS1
TACC3	ZDHHC8P1		RHOC		IRAK2
NOP14-AS1	IGLL1		RORC		P4HTM
S100P	CABIN1		ETV3		TWF2
WDR1	ELFN2		RNF187		MCM2
GLRX	SLC16A8		GATA3		RAB7A
DND1	POLR3H		PRF1		KIAA1257
STK10	SAMM50		SPOCK2		SLC26A1
RAB24	LINC00229		CALY		SLBP
PDLIM7	PRR5		APBB1		RGS12
HIST1H3D	ADM2		ZP1		LOC100129931
HIST1H4F	STT3B		CD3E		DHX15
HLA-E	XCR1		LOC100128239		RPL9
HCG27	ALS2CL		PTMS		CCT5
HSPA1B	SCAP		KRT2		DAP
LOC285847	CYB561D2		SP7		EMB
KCTD20	MINA		DYRK2		CHD1
ADAP1	NIT2		PROZ		LARP1
TTYH3	P2RY1		ZBTB1		CYFIP2
RAC1	CCNL1		LOC100289511		NPM1
BRI3	DCAF16		MIR4505		CLTB
MIR25	GRSF1		BCL11B		SLC34A1
LAMTOR4	C5orf15		REREP3		DBN1
UPK3BL	PPP2CA		LOXL1		HNRNPH1
IMPDH1	SYNPO		CIB1		TBC1D9B
GSTK1	G3BP1		SBK1		NUP153
ZYX	CNOT8		LDHD		TUBB
CTSB	CNOT6		ADAD2		FLOT1
NUDT18	SERPINB9P1		LOC100506388		FKBP5
PTK2B	PSMG4		TUSC5		STK38
REXO1L2P	GTF2H4		LOC728392		SRSF3
FAM49B	GPANK1		KCNAB3		ATP6V0CP3
SLA	ITPR3		HOXB3		PM20D2
MROH6	POLH		TRIM47		PRDM1
PARP10	SYNCRIP		MRPL54		HDDC2
SLC39A4	UBE2J1		TMIGD2		AIRN
DDX11L5	PLAGL1		TUBB4A		EIF3B
NINJ1	SCAF8		C19orf53		CHST12
WDR38	KDELR2		C19orf57		BRAT1
SLC25A25	TRA2A		LSR		FSCN1

SURF1	ZMIZ2		SMYD5		BCL7B
RXRA	LOC493754		ITGA6		STX1A
MIR4669	STAG3L3		HSPE1-MOB4		MBLAC1
MIR126	WBSCR22		BCRP3		STAG3L5P-PVRIG2P-PILRB
AGPAT2	STAG3L2		XBP1		LRCH4
FAM69B	MGC72080		IL2RB		CLDN15
CCDC183-AS1	KCP		LINC00852		ALKBH4
MAMDC4	ADCK2		ATRIP		FAM131B
FBXW5	NEIL2		EFCC1		ZNF746
ARRDC1	RHOBTB2		DNAJB14		MIR671
SOWAHD	RNF170		LEF1		BRF2
AVPR2	SNHG6		IL7R		PAG1
FLNA	FAM83H-AS1		ITK		JRK
POM121C	RPL8		RNF145		OPLAH
	VCP		BTN3A1		MAF1
	FAM120AOS		ZNF815P		HSF1
	MIR24-1		LINC00996		CYHR1
	POLE3		MIR3907		RPP25L
	RPL35		FBXO32		ZNF367
	FPGS		PTCH1		NIPSNAP3B
	SET		TRAF1		PPP6C
	PPAPDC3		RAB14		FAM129B
	GTF3C5		ADAMTSL2		ENG
	DKFZP434A062		SARDH		UCK1
	USP27X-AS1		FOXO4		WDR5
	CD52				MRPS2
	CYMP				EGFL7
	FAM19A3				RABL6
	KIAA1614				NELFB
	KISS1				EXD3
	AKR1C1				WAS
	PROSER2-AS1				BCAP31
	MRPL49				ARHGAP4
	NEAT1				HCFC1
	RNASE8				IKBKG
	CMTM5				
	LRR1				
	DIO3OS				
	MIR4513				
	FAM174B				
	C17orf97				
	FBXW9				
	SIGLEC17P				
	PROKR1				
	CD8B				
	PLAC4				
	MIR1286				
	EMID1				
	FBLN2				

	MIR3714				
	ARIH2OS				
	UBE2B				
	FAM26F				
	SNORA15				
	MIR5090				
	KLF9				
	NR5A1				
	GFI1B				
	CELP				
