# Supplementary material for: Changes of 5-hydroxymethylcytosine distribution during myeloid and lymphoid differentiation of CD34+ cells
Source: Epigenetics Chromatin. 2016 May 31;9:21. doi: 10.1186/s13072-016-0070-8 (PMC4888655; doi:10.1186/s13072-016-0070-8)
Supplement: Supplementary file 4 — 10.1186/s13072-016-0070-8 Primer used in pyrosequencing and coordinates of DNA sequence analyzed. [file 13072_2016_70_MOESM4_ESM.pdf]

Table S3: Primer used in pyrosequencing and coordinates of DNA sequence analyzed.

Primer name	Sequence	Modification	Length of the initial PCR	Coordinates of the sequence analyzed	Number of CpG assessesd
pyro AZU1 Fw	AGGTTTGTGTATAAGGGTAGT				
pyro AZU1 RcB	TCCCCCATCCTCCCACTCCCT	5' biotin	317 bp	chr19:827,797-828,112	6
pyro AZU1 Seq	GGTAGTAGTAGTTTTAGTTATAGAT				
pyro CD19 Fw	AGGTGGTATGGTGGTTAGA				
pyro CD19 RcB	CTACTCCAAAAAACTCACATTCT	5' biotin	306 bp	chr16:28,943,165-28,943,309	7
pyro CD19 Seq	GGTATGGTGGTTAGAT				
pyro FOXO1 Fw	TGTGTGTAAAATAATTTTGGTGTTAATAAT				
pyro FOXO1 RcB	ACTACAACCTCCCACTCTCTAATTCAA	5' biotin	339 bp	chr13:41,238,015-41,238,353	5
pyro FOXO1 Seq	GAAAGGTATGTTTTAAAAAGG				
pyro IRAK3 Fw	AAGGAAGGAAGGGAGAAGTTTT				
pyro IRAK3 RcB	CTAACTTTACCCCCCTTCACACCTCTAC	5' biotin	201 bp	chr12:66,582,694-66,582,816	5
pyro IRAK3 Seq	ATAAATTTTTGTAAGAAGTAATGAT				
pyro TNFRSF Fwb	AGTAGTTGGAATTGTAGGTGTGTA	5' biotin			
pyro TNFRSF25 Rc	CCAACCCTAAAAACCACTTCAA		277 bp	chr1:6,526,524-6,526,725	5
pyro TNFRSF Seq	CAAAACAAATAACTTCTCTCT				
RUNX1 Fw	AGAGGGTGGTGGGAGGATA				
RUNX1 Rcb	CAACACAACATCCCCCACAT	5' biotin	108 bp	chr21:36,258,999-36,259,106	10
RUNX1 seq	TGGTGGGAGGATAGG				
