# Supplementary material for: Changes of 5-hydroxymethylcytosine distribution during myeloid and lymphoid differentiation of CD34+ cells
Source: Epigenetics Chromatin. 2016 May 31;9:21. doi: 10.1186/s13072-016-0070-8 (PMC4888655; doi:10.1186/s13072-016-0070-8)
Supplement: Supplementary file 5 — 10.1186/s13072-016-0070-8 Publically available histone ChIP-seq data sets used. [file 13072_2016_70_MOESM5_ESM.pdf]

Table S4: Publically available histone ChIP-seq data sets used.

CB-CD34+	H3K4me1	GSM486708
	H3K4me3	GSM486709
	H3K27ac	GSM772870
	H3K27me3	GSM486704
	H3K9me3	GSM486712
	Input	GSM537662
		GSM486702

CD4+	H3K4me1	GSM772924
	H3K4me3	GSM772925
	H3K27ac	GSM772997
	H3K27me3	GSM772998
	H3K9me3	GSM772966
	Input	GSM772930

CD14+	H3K4me1	GSM1102793
	H3K4me3	GSM1102797
	H3K27ac	GSM1102782
	H3K27me3	GSM1102785
	H3K9me3	GSM1102801
	Input	GSM1102807

CD19+	H3K4me1	GSM1027296
	H3K4me3	GSM1027300
	H3K27ac	GSM1027287
	H3K27me3	GSM1160194
	H3K9me3	GSM1160205
	Input	GSM1027304
