# Supplementary material for: Changes of 5-hydroxymethylcytosine distribution during myeloid and lymphoid differentiation of CD34+ cells
Source: Epigenetics Chromatin. 2016 May 31;9:21. doi: 10.1186/s13072-016-0070-8 (PMC4888655; doi:10.1186/s13072-016-0070-8)
Supplement: Supplementary file 6 — 10.1186/s13072-016-0070-8 Primers used in qRT-PCR. [file 13072_2016_70_MOESM6_ESM.pdf]

Table S5: Primers used in RT-PCR

Gene name		Sequence
TNFRSF25	Fw	CTCACAAGCCCCTGGTTACTG
	Rc	GTTACCCACCAACTGGACGG
CD19	Fw	GGAACCTCTAGTGGTGAAGGTG
	Rc	ATCTGAGGTCCCCTTGAGGC
AZU1	Fw	GCAGAACCTGAACGACCTGA
	Rc	GAGGCAGTGGCAGTATCGTC
IRAK3	Fw	TGCAGTGTGTAGGTGACACG
	Rc	AGCATGGTTGAACGTTGTGC
FOXO1	Fw	GGATGGCATGTTCATTGAGCG
	Rc	ACTGCTTCTCTCAGTTCCTGC
